# Non-Communicable Disease (NCD) Management During Disasters and Humanitarian Emergencies: A Review of the Experiences Reported by Emergency Medical Teams (EMTs)

**DOI:** 10.3390/jpm15060255

**Published:** 2025-06-16

**Authors:** Emanuela Parotto, Flavio Salio, Martina Valente, Luca Ragazzoni

**Affiliations:** 1 Department of Anaesthesia and Intensive Care in Transplant Surgery and Major Surgery, Azienda Ospedale Università, 35121 Padova, Italy; 2 World Health Organization, Emergency Medical Teams Initiative, 1211 Geneva, Switzerland; 3 CRIMEDIM-Center for Research and Training in Disaster Medicine, Humanitarian Aid and Global Health, Università del Piemonte Orientale, 28100 Novara, Italy; martina.valente@uniupo.it (M.V.); luca.ragazzoni@med.uniupo.it (L.R.); 4 Department for Sustainable Development and Ecological Transition, Università del Piemonte Orientale, 13100 Vercelli, Italy

**Keywords:** Emergency Medical Teams (EMTs), disasters, humanitarian crisis, Non-Communicable Diseases (NCDs), chronic health needs, continuity of care

## Abstract

**Background/Objectives**: Non-Communicable Diseases (NCDs) place an excessive strain on health systems in disaster-affected settings and may lead to a parallel public health emergency lasting months or years after a disaster. Although NCDs are increasingly recognized as a major challenge in disasters and humanitarian emergencies, a dedicated and standardized response plan is missing, as well as a shortage of evidence-based guidelines for NCD management in theses contexts. Over the years, Emergency Medical Teams (EMTs) have traditionally been deployed to manage acute conditions such as trauma and infectious diseases that quickly impact health systems. However, greater attention is needed to address acute exacerbation of NCDs and to ensure continuity of care for people with chronic health needs in disasters and emergencies. **Methods**: We conducted a scoping review exploring the EMTs’ management of chronic NCDs during disasters and humanitarian emergencies, in order to identify the strategies adopted, the challenges faced, and the recommendations provided to address this health problem. The online databases PubMed, Scopus, and EBSCO were searched to identify relevant papers. **Results**: After screening the papers against the eligibility criteria, 17 publications were retrieved. Five different areas of intervention concerning EMTs and NCDs management were identified: (i) EMTs pre-departure preparation, operational time, and length of stay; (ii) EMTs staff composition and training; (iii) EMTs logistics; (iv) EMTs integration with local health services; (v) EMTs clinical data record. **Conclusions**: The findings emerging from this study showed that NCDs significantly impact disaster response in different settings, underlining the need to implement a range of EMTs activities to guarantee assistance for chronic health needs. In view of strengthening the ability of health systems to cope with the NCDs’ burden, the EMTs’ initiatives should be considered as a bridge between the support provided during the acute phase of an emergency and the continuation of care ensured by the system in its early recovery phase.

## 1. Introduction

Sudden-onset disasters can cause an extensive number of injuries and profoundly impact healthcare systems, generating a set of unique challenges that could exceed a country’s capacity to respond [1]. In such contexts, Emergency Medical Teams (EMTs) play a critical role in delivering timely and life-saving care to affected populations [2]. To support coordinated and high-quality responses, the World Health Organization (WHO) launched the EMTs Initiative and published the “Blue Book,” which defines classification and minimum standards for EMTs [2,3]. According to the WHO’s “Blue Book”, EMTs are defined as “groups of health professionals, including doctors, nurses, paramedics, support workers, logisticians, who treat patients affected by emergencies or disasters” [2]. EMTs are classified into three types based on mobility and care level: Type 1 provides outpatient and primary care; Type 2 offers inpatient acute care, including general and obstetric surgery; and Type 3 delivers complex inpatient care with intensive care capacity [2]. Specialized units may also be embedded within Type 2 or 3 teams or local facilities, focusing on services like rehabilitation, mental health, reproductive health, or technical support [2,3].

In the past few decades, EMTs have traditionally been deployed to manage acute conditions, such as trauma and infectious diseases, that can quickly impact the primary surge capacity of the affected health systems [4,5,6]. However, less attention has been devoted to preparedness for the secondary and recovery phases of disaster response, which involve expanding operational capacity to ensure continuity of care [7,8]. This is particularly critical for the management of chronic Non-Communicable Diseases (NCDs), which require sustained treatment, regular monitoring, and uninterrupted access to medications. This gap in continuity of care is especially concerning in Low- and Middle-income countries (LMICs), where vulnerable populations are disproportionately affected by NCDs. If these conditions are not adequately managed throughout the recovery phase, they can escalate into long-term public health emergencies that further burden already fragile health systems [8,9]. This risk is heightened in LMICs, where NCDs burden and humanitarian crises often co-occur [10].

Although it is recognized that NCDs represent an increasing global challenge during disasters and humanitarian emergencies, standardized emergency response plans and evidence-based guidelines remain limited [10]. Establishing EMTs focused on providing long-term primary healthcare services, including specialized care for NCDs, could help mitigate the strain on health systems. The WHO’s “Blue Book” outlines minimum standards for chronic NCD care across EMT types, but more guidance is needed for effective long-term management [10].

The aim of this literature review was to examine how Emergency Medical Teams (EMTs) addressed the management of NCDs during disasters and humanitarian emergencies, with a particular focus on the strategies adopted, the challenges encountered, and the recommendations proposed to improve care delivery.

## 2. Materials and Methods

This scoping review was conducted following the Joanna Briggs Institute (JBI) guidelines [11] and the PRISMA Extension checklist for Scoping Reviews (PRISMA-ScR) [12]. The research questions, the frame search, and the inclusion criteria were developed using the PCC elements with ‘P’ denoting population, ‘C’, the concept, and ‘C’, the context, according to the JBI’s methodological approach to conducting scoping reviews [11] (Supplementary Table S1). The specific research questions were as follows: (i) What strategies did EMTs adopt to manage NCDs during disasters and humanitarian emergencies? (ii) What challenges did EMTs face in managing NCDs? (iii) What recommendations did EMTs provide for addressing NCDs during such events? The search strategy was built around the key terms and their variations in “Emergency Medical Teams”, “Disasters”, and “Non-Communicable Diseases”, and executed in the databases PubMed, Scopus, and EBSCO (CINAHL, Medline, PsycINFO) in December 2022. MeSH terms and Subject terms were used. The three groups of keywords were combined by using the Boolean operators AND and OR. A search for references that appeared to be pertinent was also carried out. In order to optimize our search on such an underexplored topic, we decided to include in this review both EMTs WHO classified and those not WHO classified. The main eligibility criteria for articles to be included in the study were the following: (i) the study concerned Emergency Medical Teams, including both WHO classified and not-WHO classified EMTs; (ii) the study included chronic NCDs in the list of diseases managed by EMTs during their deployment or the study was specifically focused on EMTs management of NCDs; (iii) the study was written in English. In order to obtain a comprehensive overview of the subject, no restrictions were applied regarding the study type or year of publication. Articles were therefore excluded whenever they were not focused on EMTs or did not report chronic NCD management in the context of EMT activities.

Disaster types were recorded as reported in the original studies and both natural and human-made events were included. EMTs’ classifications were extracted based on the WHO “Blue Book” framework [2]; however, not all EMTs described in the included studies were formally classified under the WHO system. In cases where classification was unclear or not provided, we reported the available characteristics and deployment details to the extent possible. Given the heterogeneity in how disaster types and EMTs were described across studies, complete standardization was not feasible. We adopted a broad definition of NCDs in line with the current literature and WHO guidance, which includes cardiovascular diseases, cancers, chronic respiratory diseases, and diabetes, as well as mental health conditions and injuries. This inclusive approach reflected the reality that, in many emergency and humanitarian contexts, EMTs are required to manage a wide spectrum of chronic and long-term conditions beyond the traditional four major NCDs (cardiovascular diseases, cancers, chronic respiratory diseases, and diabetes) [10]. Although NCDs can have acute presentations [13], the majority demand continuous care, regular monitoring, and consistent access to medications, which are especially difficult to maintain when health systems are disrupted [14,15]. Notably, NCDs account for 75% of global deaths, with 82% of premature deaths occurring in LMICs [15]. Given this substantial burden and the growing recognition that emergency responses must address both acute and chronic health needs, we adopted a broad inclusion approach to better capture the evolving role of EMTs in managing NCD-related conditions during disasters and humanitarian emergencies. The search and screening of titles, abstracts, and full texts were performed by authors EP and MV, against the agreed inclusion criteria; disagreements between reviewers were resolved by consensus.

Data was extracted from the retrieved articles and recorded into a prespecified data extraction table (Supplementary Table S2). Extracted information was analyzed to explore EMTs’ activities with respect to NCD management, with a major focus on EMT type and clinical care technical standards adopted during the deployments in different disaster settings. Specifically, the following data was extracted: (i) study authors and publication date, (ii) study title and study aim, (iii) study type and design, (iv) disaster type and country of occurrence, (v) EMTs type, (vi) EMTs time of deployment and length of stay, (vii) EMTs staff and training, (viii) EMTs equipment, (ix) type of NCDs encountered, (x) actions taken and strategies adopted to manage NCDs, (xi) challenges faced by EMTs in relation to NCDs management, (xii) recommendations provided by EMTs to improve NCDs management. A narrative synthesis approach based on thematic analysis was used to analyze and synthesize the findings across the included studies. This method was chosen due to the heterogeneity of study designs, interventions, and outcomes, which made a meta-analysis not feasible. Through thematic analysis, key patterns and themes related to EMTs’ strategies adopted, challenges faced, and recommendations provided in relation to NCD management were identified and synthesized. The process was led by the primary author EP and iteratively discussed with the co-author MV to ensure consistency, reflexivity, and shared interpretation of the data. The World Health Organization’s “Classification and Minimum Technical Standards for Emergency Medical Teams” (the WHO Blue Book) [2] was used as a guiding framework to map and interpret the findings in relation to the EMT organization and NCD care provision during humanitarian emergencies.

## 3. Results

**Figure 1:**
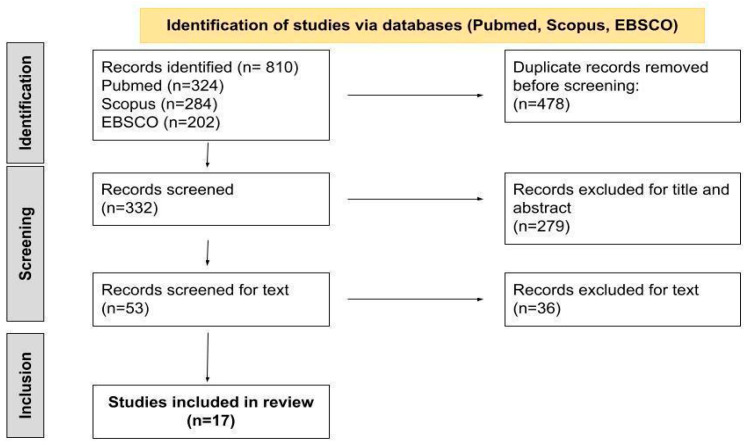
Identification of studies from the database search (PubMed, Scopus, EBSCO).

A total of 810 records were identified from the database search. Duplicates (n = 478) were removed, leaving 332 articles to be judged for relevance. Of those, 279 were excluded, leaving 53 records whose full text was screened. After evaluation, 17 studies were included in the review (Figure 1).

Among the studies included, seven studies were published in 2022 [16,17,18,19,20], two in 2021 [21,22], one in 2020 [23,24], two in 2019 [25,26], one in 2017 [27], one in 2016 [28], one in 2013 [29], and two in 2007 [30,31]. Of the included studies, 12 focused on specific disaster events, such as the 2004 tsunami in Indonesia [31], the Syrian civil war [28], major earthquakes in China and Pakistan [29,30], Typhoon Haiyan in the Philippines [26,27], Cyclone Idai in Mozambique [18,21], Hurricane Dorian [25], the West Japan Heavy Rain [17,19], and the COVID-19 pandemic [22]. The remaining five studies took a more general perspective: one focused broadly on earthquakes [23], one examined multiple disaster types in South Sudan (including conflicts, floods, and disease outbreaks) [16], and three aimed to develop general standards for EMT operations in disaster settings [20,23,32]. In terms of methodological approaches, 13 studies were based on descriptive statistical analyses [16,17,18,19,21,22,25,26,27,28,29,30,31], one was a two-phase mixed-method study using a Delphi methodology and a cross-sectional survey [32], one was a desk-based study [22], one was a design plan study [20], and one was a discussion paper [24]. The main characteristics of the studies included in this review are reported in Table 1.

**Table 1:** Main characteristics of the studies included in the review.

Studies Included						
First Author	Year of Publication	Title	Methodology	Aim	Disaster Setting	Disaster Time of Occurrence
Fernald [30]	2007	The Mobile Army Surgical Hospital Humanitarian Assistance Mission in Pakistan: The Primary Care Experience	Cross-sectional study	To describe the experiences during the deployment of a mobile army surgical hospital	Pakistan earthquake	8 October 2005
Guha-Sapir [31]	2007	Patterns of chronic and acute diseases after natural disasters—a study from the International Committee of the Red Cross field hospital in Banda Aceh after the 2004 Indian Ocean tsunami	Cross-sectional, record-based study	To assess the pattern of diseases in the immediate aftermath of the 2004 Indian Ocean tsunami	Indian Ocean tsunami	December 2004
Hung [29]	2013	Disease pattern and chronic illness in rural China: the Hong Kong Red Cross basic health clinic after the 2008 Sichuan earthquake	Cross-sectional records-based study	To identify the health needs and chronic disease prevalence of rural Chinese following a major earthquake	Sichuan earthquake, China	12 May 2008
van Berlaer [28]	2016	A refugee camp in the center of Europe: clinical characteristics of asylum seekers arriving in Brussels	Descriptive cross-sectional study design	To describe the demographic and clinical characteristics of asylum seekers who arrived in a huddled refugee camp in Brussels	Syrian civil war and Syrian exodus	Summer 2015
McDermott [27]	2017	Management of Diabetic Surgical Patients in a Deployed Field Hospital: A Model for Acute Non-Communicable Disease Care in Disaster	Descriptive analysis	To improve the care of diabetic patients in humanitarian settings by exploring a case study of NCD management in a surgical field hospital	Typhoon Haiyan (Yolanda), Philippines	8 November 2013
Dunne-Sosa [25]	2019	The Hidden Wounds of Hurricane Dorian	Field Report	To report the mission of the HOPE Emergency Response Team	Hurricane Dorian, Bahamas	1 September 2019
van Berlaer [26]	2019	Clinical Characteristics of the 2013 Haiyan Typhoon Victims Presenting to the Belgian First Aid and Support Team	Cross-sectional study	To document the demographics, complaints, comorbidities, diagnoses, diagnosis categories, and management of typhoon victims who sought medical assistance in a field hospital of an international EMT, and to formulate recommendations for future relief operations	Typhoon Haiyan (Yolanda), Philippines	8 November 2013
Bartolucci [23]	2021	Decision Support Framework for Deployment of Emergency Medical Teams After Earthquakes	Desk-based study	To enhance disaster managers’ literacy and to provide a framework that will assist those responsible for deploying and/or accepting EMTs in making informed decisions on the deployment of emergency medical teams after an earthquake	Earthquakes	Not specified
McMaster [24]	2020	Integrating specialist ophthalmic services into emergency medical teams	Discussion paper	To describe the importance of increasing specialist ophthalmic services within emergency medical teams	Conflicts (not specified), Earthquakes, infectious disease outbreaks	Conflicts (Timor-Leste and others not specified), Earthquakes (Japan in 2011 and Nepal in 2015), Ebola (West Africa, 2013–16) and measles outbreaks (Pacific region, 2019)
Ladeira [21]	2021	PT EMT—Portuguese Emergency Medical Team Type 1 Relief Mission in Mozambique	Descriptive analysis	To report the mission of the PT EMT type 1 in Mozambique	Cyclone Idai	15 March 2019
Dulacha [16]	2022	Use of mobile medical teams to fill critical gaps in health service delivery in complex humanitarian settings, 2017–2020: a case study of South Sudan	Descriptive analysis	To analyze the key achievements of emergency mobile medical teams (eMMT) in disaster settings of South Sudan	Conflicts, floods, famine, and disease outbreaks in South Sudan	2017–2020
McMaster [20]	2022	Designing a Mobile Eye Hospital to Support Health Systems in Resource-Scarce Environments	Discussion paper	To propose a design plan for a mobile eye hospital to support health systems between the initial emergency response and recovery of health infrastructure in resource-scarce environments of low- and middle-income countries.	Not specified	Not specified
Foo [32]	2022	Establishment of disaster medical assistance team standards and evaluation of the teams’ disaster preparedness: An experience from Taiwan	Delphi study (Phase 1)/Cross-sectional study (Phase 2)	To develop localized Disaster Medical Assistance Teams (DMATs) standards for Taiwan by referring to EMT type I standards, and to further evaluate the disaster preparedness of Taiwan’s DMAT	Chi-Chi earthquake	September 2019
Chimed Ochir [19]	2022	Emergency Medical Teams’ Responses during the West Japan Heavy Rain 2018: J-SPEED Data Analysis	Descriptive epidemiology study	To better understand the health problems during floods and heavy rain disasters	West Japan Heavy Rain	8 July–11 September 2018
Sacchetto [18]	2022	Italian Field Hospital Experience in Mozambique: Report of Ordinary Activities in an Extraordinary Context	Descriptive analysis	To report the mission of the EMT2-ITA in Mozambique, raising interesting points of discussion regarding the impact of timing on the mission outcomes, the operational and clinical activities in the field hospital, and the great importance to integrate local staff into the team	Cyclone Idai, Mozambique	15 March 2019
Tachikawa [22]	2022	Mental health needs associated with COVID-19 on the diamond princess cruise ship: A case series recorded by the disaster psychiatric assistance team	Descriptive analysis	To assess the clinical characteristics of patients with acute mental health needs on the quarantined ship Diamond Princess and recommend evidence-based measures for disaster mitigation	COVID-19 Pandemic, Japan	9–21 February 2020
Yumiya [17]	2022	Prevalence of Mental Health Problems among Patients Treated by Emergency Medical Teams: Findings from J-SPEED data regarding the West Japan Heavy Rain 2018	Descriptive analysis	To examine how mental health needs are accounted for in the overall picture of disaster relief, and how they change overtime	West Japan Heavy Rain	8 July–11 September 2018

Findings were detailed below across three different domains: (1) NCD management during EMT deployment (Table 2); (2) EMT characteristics (Table 2); and (3) recommendations for improving NCD care (Table 3).

**Table 2:** Emergency Medical Teams (EMTs) characteristics and Non-Communicable Diseases (NCDs) management as reported in the studies included in the review.

Emergency Medical Teams (EMTs) and Non-Communicable Diseases (NCDs) Management					
First Author/ Year of Publication	EMTs Type	EMTs Clinical Staff	EMTs Equipment (Related to NCDs)	NCDs Registered	Challenges Reported (Related to NCD Treatment)
Dulacha/2022 [16]	Type 1 mobile	Epidemiologists, clinicians or doctors, nurses, laboratory specialists, nutritionists, health promotion experts, and public health officers	Emergency health kits Laboratory sample collection kits	Chronic conditions not specified	Absence of strategies to ensure the continued provision of services for the chronic conditions initially managed during the mobile outreach
McMaster/2022 [20]	EMT Specialized in eye diseases (a mobile eye hospital)	Ophthalmologists, ophthalmic assistants, nurses, and anesthesiologists	Examination equipment with visual acuity charts, a portable slit lamp, indirect ophthalmoscope, tonometer, fundus lenses, compact A/B ultrasound scanner, autorefractor, sphygmomanometer, and expendable supplies such as eye drops and reagents for estimating urine sugar concentration	Chronic eye diseases (including diseases caused by NCDs)	Not specified
Chimed Ochir/2022 [19]	Not specified	Not specified	Not specified	Cardiovascular diseases, disaster stress-related symptoms	Not specified
Sacchetto/2022 [18]	Type 2	58 healthcare professionals (29 medical doctors, including two team leaders and one deputy team leader; 27 nurses; one x-ray technician; and one midwife)	Not specified	Cardiovascular, neurologic, and respiratory diseases	Only a few patients with specific disaster-related injuries: → many patients come to the field hospital for routine medical care
Yumiya/2022 [17]	Type 1 and 2	Not specified	Not specified	Mental health problems and disaster stress-related symptoms	Not specified
Tachikawa/2022 [22]	Disaster Psychiatric Assistance Team (DPAT)	Fifty-five members of 12 DPAT groups	Psychological advice and Psychiatric assistance	Mental health disorders	Not specified
Ladeira/2021 [21]	Type 1 mobile	Two-team rotation (28 elements each). Doctors of different specialties (i.e., intensive care, internal medicine, pediatrics, surgery, obstetrics, and infectious diseases); specialized nurses in critically ill patients, or emergency and disaster; prehospital technicians; psychologists; x-ray technicians; pharmaceuticals	X-Ray, in addition to the standard equipment	Low back pain, headache, and gastritis	The World Health Organization Minimal Data Set register is insufficient to allow an adequate classification of all the NCDs managed during the EMT deployment
Foo/2021 [32]	DMAT (Disaster Medical Assistance Teams) type 1 fixed	1:2:2 ratio of physicians:nurses:logisticians	Ultrasound services have been added to the standard services	Emergency chronic disease care	Not reported
Bartolucci/2020 [23]	Type 1, 2, and 3	Not specified	Not specified	Chronic health conditions	Difficulties in standardizing the EMTs’ time of deployment Unpredictability of disasters, logistical complications, and local protocols and procedures that can affect the EMTs’ registration and location assignments
McMaster/2020 [24]	Type 1, 2, and 3	Specialist ophthalmology units capable of integrating into type 1, 2, and 3 EMTs	Not specified	Not specified	Not specified
van Berlaer/2019 [26]	Belgian First Aid and Support Team (B-FAST)	Volunteer team comprising 5 physicians (1 surgeon, 3 anesthesiologists trained in emergency medicine, and 1 pediatrician); 15 skilled nurses	Preconfigured interagency emergency health kits (IEHKs)	Diabetes, hypertension, asthma, and mental health disorders	The widely used basic IEHK, does not contain sufficient medication refills for patients, so B-FAST provided, by their own means, refills of medication and distribution of materials like beta-blockers, inhalers, and urine ketone strips Follow-up or referral of patients
Dunne Sosa/2019 [25]	HOPE Emergency Response Team	Medical volunteers	Insulin needles, hygiene kits	Chronic diseases such as diabetes, hypertension, and cancer	Not specified
McDermott/2017 [27]	Type 2	Two consecutive acute trauma surgery teams, 1 internal medicine physician per team, 1 pharmacist per team, no nursing staff with a primary specialty of patient inward	Blood glucose monitoring, dipstick Urinalysis, fast-acting insulin, metformin	Diabetes	Local medical supplies depleted quickly: →people with diabetes found that they had no medication and limited means of obtaining renewed supply The medication available was limited →normal regimes were not available →people had to change to unfamiliar drugs Prescriptions were not necessarily available along with medical records →reliance was made on diabetic patients memorizing their regular medications
Van Berlaer/2016 [28]	Field Hospital (Médecins du monde, MdM)	400 certified physicians, nurses, pharmacists, logisticians, and interpreters. MdM registered all volunteers and verified their diplomas and license to work in Belgium. An outpatient assistance team with a physician, a nurse, and an interpreter provided on-the-spot healthcare for patients not able to leave their tents, or referred them to the field hospital for further care when necessary	Not reported	Respiratory diseases, skin, digestive diseases, hypertension, diabetes, asthma, epilepsy, mental health issues	Not reported
Hung/2013 [29]	Hong Kong Red Cross (HKRC) basic healthcare clinic	HKRC medical teams are composed of seven doctors, six nurses, and one senior health coordinator, all providing basic healthcare to villagers	Not reported	Musculoskeletal, respiratory, gastrointestinal problems, and a high prevalence of hypertension	Management of chronic diseases was an important issue
Guha-Sapir/2007 [31]	Red Cross field hospital	Not reported	Not reported	Respiratory diseases, hypertension, diabetes, and acute manifestation of chronic diseases (e.g., asthma), mental diseases, chronic musculoskeletal disease, headache, gastroesophageal reflux, cerebrovascular accidents, renal failure, myocardial infarction	
Fernald/2007 [30]	Mobile Army Surgical hospital (MASH)	Surgery team implemented with two family medicine physicians, one pediatrician, and one internist	Not reported	Chronic musculoskeletal disease, headache, gastroesophageal reflux, cerebrovascular accidents, renal failure, myocardial infarction, respiratory failure	After the first month of the mission, the surgical patient load decreased, whereas primary care increased to 90% of patient encounters →delay in establishing a clear, concise plan for primary care

**Table 3:** Recommendations on Emergency Medical Teams’ (EMTs) management of Non-Communicable Diseases (NCDs) during disasters and humanitarian emergencies, as emerged from the included papers.

Non Communicable Diseases (NCDs) Mana	
Emergency Medical Teams (EMTs)	Suggestions
Pre-departure preparation	Ensure that patients with chronic conditions are monitored and their medication is maintained during the disaster [21]
	Stockpile disaster-response equipment and drugs in strategic areas in disaster-prone regions to guarantee that proper EMT equipment is promptly available [28]
	Align with the national pharmaceutical formulary of the disaster-affected host nation to guarantee proper EMT equipment and an adequate pharmacological load [29]
	Define clear operational guidelines on NCDs field management and on patients’ follow-up [29]
	Medical responders need to be aware of the potential pre-existing disease burden in the community, with the possible exacerbation in post-disaster situations [31]
Operational time and length of stay	Become operative two or more weeks after a disaster requires being more prompt in ensuring elective activities aimed at maintaining the ordinary healthcare capacity of the affected country [20]
	Follow a recovery approach based on different time periods in order to guarantee assistance for health needs that arise at different times in disasters aftermath [19]
	Provide a semi-permanent service between the initial emergency response and the re-establishment of local health services delivery [22]
	Ensure a continuous services provision system for patients when an EMT exit strategy is planned [19]
	Ensuring continuity of care for longer than the initial two weeks after the disaster onset [27]
Staff composition and training	Include internal medicine specialists and nurses familiar with inpatient management of chronic diseases to be deployed in chronic condition management, such as diabetes [29]
	Including family medicine physicians and internists to ensure primary care for the affected population [32]
	Train staff to be deployed in chronic conditions management, mental health disorders, and psychosocial problems management [18,19]
	Deployed health professionals should be trained in specific conditions management, such as ocular diseases [26]
	Medical teams should be prepared for acute presentations of chronic illnesses [33]
	Psychiatric care should be anticipated for both disaster-related and pre-disaster patients [33]
	Compassion fatigue and staff burnout must be anticipated. In prolonged missions, 1 day of rest per week for staff members is essential. Translators are especially vulnerable to mental fatigue [32]
	EMTs addressing mental health issues are essential services for the maintenance of public health during crisis situations [24]
Equipment	Enhance medication inventories to include a range of common drugs for chronic diseases and stock pharmacies adequately [27,29,32]
	Consider the addition of X-Ray (EMTs type 1) in the standard equipment to improve the diagnosis capacity [23]
	Consider the addition of ultrasound (EMTs type 1) in the standard equipment to improve the diagnosis capacity [34]
	Consider the addition of analgesic drugs in the standard equipment to better address the needs of vulnerable populations [23]
	The cold chain system is a necessary and basic requirement to store medications such as insulin [27,34]
Integration with local health staff	Working in integration and not in overlap with local health services [16,18,20,23]
	The collaboration with community health workers, community-based organizations, local health facilities, and non-governmental organizations (NGOs) is a key element to ensure the continuous treatment of patients with chronic illnesses [16,18,29,31]
	The collaboration with local health staff is a key element to promote the health education of the local population, including education on emergency preparedness [27,28]
	The collaboration with local health staff is a key element to guarantee the sustainability of EMT interventions [18]
	The integration of the local staff in the team composition during the rotation of the personnel allowed, on one side, to limit the number of professionals coming from the EMTs’ country of origin and, on the other side, to ensure an effective training of local health workers [20]
	EMTs can work synergistically to achieve better outcomes [34]
Data collection and reporting system	An adequate data collection system is crucial to report EMTs’ clinical activities, in order to avoid underestimation of chronic health conditions [21]
	An adequate data collection system is crucial to facilitate the reintegration of patients into the local health system [29]
	The implementation of digital health services allows EMTs to capture the relevant data from the catchment populations where they are conducting their outreach [23]
	A more precise identification of NCDs in the WHO EMTs Minimum Data Set (MDS) should be considered, in order to guarantee a more precise identification of EMTs’ clinical activities [23]
Other	The government should formulate a system to provide EMT members with adequate insurance when deployed (they work under safety and security threats) [34]
	EMTs should receive adequate financial support (2021) [34]
	EMTs should educate patients with chronic illnesses on emergency preparedness [34]

### 3.1. NCDs Management

#### 3.1.1. NCDs Reported

Four of the seventeen reviewed papers focused specifically on chronic NCDs, addressing diabetes during Typhoon Haiyan [27], and chronic diseases post-earthquake in China [29], Indonesia [31], and among Syrian refugees [28]. The other 13 papers had a broader approach, which included the management of chronic NCDs in the context of all the clinical activities performed by EMTs during their deployment [17,18,19,20,21,22,23,24,25,26,30,32]. Three articles generically referred to chronic conditions [16,23] or primary healthcare needs [30], four discussed mental health and disaster stress-related disorders [17,19,22,26], and seven mentioned specific chronic conditions, including cardiovascular, respiratory, metabolic, neurological, musculoskeletal, dental, and digestive diseases [18,19,21,26,28,32]. Finally, two papers were specifically focused on ocular disease management [20,24] (Table 2).

#### 3.1.2. Challenges Faced

Several authors highlighted the lack of adequate chronic care during disasters, underlining gaps in policy and research [29], the shortage of EMT models addressing NCDs [18,19,27,29,30], and the lack of specialization in psychosocial support and mental healthcare [16]. Key challenges in NCDs management included shortages of essential medications [26,27,29], missing medical records [21,27], absence of continuity-of-care strategies [16], and limited integration with local health systems [29].

#### 3.1.3. Actions Taken

The health measures adopted by EMTs to manage NCDs included pharmacological treatments administrations, such as short-acting insulin and hypoglycemic [27], analgesic drugs [21,26], bronchodilators [26], life-saving medications [25], and surgical care for diabetic wounds [27], and ocular diseases [20]. EMTs also provided outpatient consultations, health promotion services [16], and first aid training for the disaster-affected population [25].

### 3.2. EMTs Characteristics

The main characteristics of the EMTs reported in the papers included in this review are detailed in Table 2.

#### 3.2.1. Type

Seven studies referred to EMTs classified in accordance with the WHO minimum standard. Two of them specifically considered EMTs type 1, one fixed [21] and the other one mobile [16], while three other papers focused on EMTs type 2 [18,26,27]. Moreover, two papers of this group described activities led by both EMTs type 1 and 2 [17] or by EMTs type 1, 2, 3 [23]. The remaining ten papers included in this review referred to a more heterogeneous group of EMTs. One of these papers analyzed data reported by 85 EMTs [19] without providing a specific reference to the different EMT types. Two other studies proposed a model of specialized EMT focused on ophthalmic services [20,24]. Three papers described activities performed in the context of field hospitals [28,30,31]. In addition, four papers variously referred to the assistance provided by a basic health clinic [29], a Disaster Medical Assistance Teams (DMATS) [32], a Disaster Psychiatric Assistance Team (DPAT) [22], and by emergency medical teams focused on chronic health needs [25].

#### 3.2.2. Staff Composition

Several studies highlighted the varied composition of EMTs’ clinical staff. Type 1 teams reported by Ladeira et al. included doctors of different specialties (such as intensive care, internal medicine, pediatrics, surgery, obstetrics, and infectious diseases), specialized nurses in critically ill patients, and psychologists [21]. Mobile EMTs described by Dulacha et al. comprised doctors and nurses alongside epidemiologists, lab specialists, nutritionists, and health promotion experts [16]. Among Type 2 EMTs, staff profiles included internal medicine physicians, pharmacists, anesthesiologists with experience in emergency medicine, x-ray technicians, nurses, and midwives [18,26,27]. Other EMTs not classified by WHO also featured diverse configurations, such as the Disaster Psychiatric Assistance Team aboard the Diamond Princess during the COVID 19 pandemic, composed of specialized in mental health and psychosocial support [22], and a Mobile Army Surgical Hospital staffed with emergency and family medicine doctors and an internist [30].

#### 3.2.3. Operational Time, Length of Stay, and Opening Hours

The EMTs’ operations varied widely due to logistical challenges, disaster type, and registration processes [23]. Reported times ranged from 24 to 48 h [16], to six days [27], eight days [26], and up to two weeks [18,21]. The EMTs’ length of stay described in the papers retrieved showed a certain degree of variability [17,18,19,21,26,27,29,30], ranging from a minimum of 4 days [26] to a maximum of four months [30]. Only a few papers reported information concerning the EMTs’ opening hours. Van Berlaer et al. described a field hospital providing 24/7 ambulatory healthcare and psychosocial support for refugees [28], while Fernald et al. reported that the MASH Primary care service operated 12 h per day, 7 days per week [30].

#### 3.2.4. Equipment

Six papers reported information concerning the EMTs’ medical and surgical equipment and provided specific details regarding NCD treatment [16,20,21,26,27,29]. In particular, the use of antidiabetic medications (short-acting insulin and metformin) [27], analgesic drugs [21,26], and bronchodilators [26] was commonly noted. Moreover, the provision of medical devices to monitor urine glucose levels and blood pressure measures [27,29], the utilization of surgical kits to manage diabetic wounds [27], and to guarantee specialized ophthalmic care [20] were indicated as useful tools to address the health needs of vulnerable populations. The integration of diagnostic tools such as X-ray machines was also highlighted as a valuable enhancement for mobile EMTs in disaster settings [21].

#### 3.2.5. Data Collection

Tachikawa and coll. used an anonymized database known as the Japan Surveillance in Post-Extreme Emergencies and Disasters (J-SPEED). The J-SPEED recording process involved a checklist in the style of the World Health Organization mini data set (WHO-MDS) for EMTs, but it was exclusively focused on mental health events [22]. Gerlant van Berlaer et coll. reported that all the professionals operating in the field hospital to assist refugees were trained in data collection procedures [28].

#### 3.2.6. Patients’ Referral

Van Berlaer et coll. reported that refugees requiring emergency care, laboratory tests, medical imaging, or hospitalization were transferred from the field medical hospital inside the refugee camp to governmental hospitals located in the area [28]. The EMTs described by Hung et coll. adopted the policy to refer chronic conditions that require long-term treatment to the local healthcare providers [29].

### 3.3. Recommendations to Improve EMT-Related Management of NCDs

Although the majority of papers included in this review were not specifically focused on NCDs, relevant suggestions regarding the management of these health conditions emerged from the findings. The strategies proposed concerned five different areas of intervention: (i) EMTs’ pre-departure preparation, time of deployment, and length of stay; (ii) EMTs’ staff composition and training; (iii) EMTs’ equipment; (iv) EMTs’ integration and coordination; (v) EMTs’ clinical data record.

#### 3.3.1. Pre-Departure Preparation, Time of Deployment, and Length of Stay

Pre-departure preparation was reported as essential to define clear operational guidelines on NCDs field management and on patients’ follow-up [27]. The team composition must be adapted based on the nature of the emergency being responded to [16], and the EMTs’ pharmacological load should be established in alignment with the national formulary of the disaster-affected host nation [27]. Moreover, disaster-response equipment and drugs should be stockpiled in strategic areas in disaster-prone regions in order to guarantee that equipment is promptly available [26].

Although most papers did not report specific EMT deployment timelines for NCD management, four provided information on deployment timing in relation to addressing chronic conditions. Sacchetto et al. underlined that EMTs becoming operative two or more weeks after a disaster event are mainly committed to ensuring elective activities aimed at maintaining the ordinary healthcare capacity of the affected country [18]. Yumuya et al. highlighted the need to ensure a continuous service provision system for patients when an EMT exit strategy is planned [17]. McMaster et al. emphasized the exigency to provide a semi-permanent service between the initial emergency response and the reestablishment of local health services delivery, due to the fact that non-disaster-related events increased as time passed after the disaster [20]. Dunne-Sosa et al. pointed out that medical teams need to stay longer than two weeks after a disaster to properly care for patients with chronic conditions [25].

#### 3.3.2. Staff Composition and Training

The papers retrieved pointed out that EMTs must include sufficient primary care personnel, medications, and supplies to ensure primary care services [30,31,32]. It was underlined that EMTs’ clinical staff should include internal medicine specialists or equivalent, general and emergency physicians, diabetes and hypertension specialists, psychologists, and nurses familiar with inpatient management of chronic diseases [27,28]. Moreover, disaster mental health specialists were identified as essential components of EMT personnel to face the health needs of the assisted population [22]. Several Authors underlined that EMTs, health professionals, should be trained on chronic conditions management, such as diabetes, hypertension, ocular diseases, mental health disorders, and psychosocial problems [17,20,27].

#### 3.3.3. Equipment

With regard to equipment, some of the retrieved papers underlined that EMTs’ medication inventories should be enhanced to include a range of common drugs for chronic diseases [27]. With regard to EMT type 1, it was proposed to add analgesic drugs, X-ray [21], and ultrasound devices [32] to the standard equipment in order to better address the health needs of the vulnerable people. The presence of a cold chain system was recognized as a necessary and basic requirement in order to store medications for chronic health conditions, such as insulin [25,32].

#### 3.3.4. Integration and Coordination

The importance of working in integration and not in overlap with local health services was underlined by several authors [16,18,21,26,29]. The collaboration with community health workers, community-based organizations, and local health facilities was recognized as a key element to ensure the continuous treatment of patients with chronic illnesses [16,25,27,30,31], to promote the health education of the local population [25,26], and to guarantee the sustainability of interventions [16,25]. The integration of the local staff in the team composition during the rotation of the personnel allowed, on one side, to limit the number of professionals coming from the EMTs’ country of origin and, on the other side, to ensure an effective training of local health workers [18]. The presence of a synergistic and coordinated work of different emergency medical teams (e.g., governmental and private teams) was recommended as a useful strategy to achieve better outcomes in disaster aftermath [32]. Moreover, working closely not only with local healthcare providers but also with Non-Governmental Organizations (NGOs) was suggested as essential for maintaining continuity of care [30].

#### 3.3.5. Data Collection and Reporting System

From a general perspective, an adequate data collection was reported as crucial to report EMTs’ clinical activities, in order to avoid underestimation of chronic health conditions [19] and to facilitate the reintegration of patients into the local health system [27]. The development of a standardized template to prospectively collect and subsequently analyze and report health data was considered a relevant tool to significantly contribute to humanitarian emergency management [28]. The implementation of digital health services was suggested as a strategy for mobile EMTs to capture the relevant data from the catchment populations where they are conducting their outreach. Moreover, a more precise identification of NCDs in the WHO EMTs Minimum Data Set (MDS) should be considered, in order to allow a more precise identification of EMTs’ clinical activities [21].

#### 3.3.6. Other Recommendations

In order to ensure continuity of care for chronic conditions, some authors suggested measures aimed to facilitate access to prescription drugs for the population affected, and to implement health education programs for patients with chronic illnesses [25].

## 4. Discussion

The aim of this literature review was to examine how Emergency Medical Teams (EMTs) addressed the management of NCDs during disasters and humanitarian emergencies, with a particular focus on the strategies adopted, the challenges encountered, and the recommendations proposed to improve care delivery. This focus aligned with the increasing recognition of NCDs as a critical component of humanitarian health response [33,34]. The need to prioritize this global health challenge emerged at the 2022 World Health Assembly, where Member States requested WHO to provide technical assistance to strengthen NCDs in humanitarian crises. In response, WHO established a joint program of work between the NCDs Department and WHO Health Emergencies Program (WHE), including a review of WHO support for NCDs programming in recent graded emergencies, development of an operational manual on NCDs in emergencies, and a series of regional workshops on NCDs in emergencies [14]. In parallel, the United Nations High Commissioner for Refugees [35,36] increasingly recognized the importance of integrating NCD care into refugee health programs and issued technical guidance for managing chronic conditions in displacement settings [35,36]. However, these efforts have yet to be fully translated into operational standards for Emergency Medical Teams (EMTs). Integrating the emerging NCDs guidance into EMT deployment models would enhance preparedness, ensure continuity of care, and improve outcomes for individuals with chronic diseases.

This evolving policy landscape is mirrored in the growing body of academic literature on NCDs in humanitarian emergencies, which reflects increasing recognition of the complexity and urgency of this challenge. As an example, Ngaruiya et al. conducted an extensive review of chronic NCDs in disaster settings and underscored the disproportionate burden carried by populations in low- and middle-income countries (LMICs), as well as the lack of targeted intervention models and clinical guidance for NCDs management during humanitarian crises [33]. Similarly, Leff et al. identified successful NCD-related interventions and emphasized the importance of early risk assessment, healthcare worker training, integration with local health services, and adaptive implementation strategies. However, both studies also exposed the paucity of evidence concerning preparedness and mitigation efforts and pointed to a general absence of structured frameworks for NCD response in emergency contexts [34]. While the aforementioned reviews offer crucial insights into the broader challenges of NCD care, our work addressed a critical gap by focusing on how EMTs can be better equipped, structured, and integrated to deliver both acute and long-term NCD care.

Our findings highlighted significant heterogeneity across the included studies in terms of geographic context, disaster type, EMT configuration, and health system integration, all of which inherently affected how EMTs function during deployment. Among the 17 papers reviewed, 12 focused on specific disaster contexts across diverse geographic settings, each marked by unique logistical, infrastructural, and health system challenges [17,18,19,21,22,25,26,27,29,30,31]. Regional and contextual variation was also evident in the composition and capabilities of EMTs. Teams varied from highly specialized units, such as those delivering ophthalmic care [24], to more generalist field hospitals supporting asylum seekers [28]. Moreover, operational time and length of stay ranged widely depending on local access, regulatory processes, and disaster severity [16,18,21,30]. Differences in staff composition and training also reflected contextual needs. Some teams included specialists in internal medicine or mental health [22,26,28], while others lacked personnel trained in chronic disease management [27]. Despite these context-specific insights, the heterogeneity of the studies precluded generalization across regions or disaster types. Rather, this diversity underscores the need for flexible, context-responsive EMT models. Importantly, this variability also reflects the distinct challenges posed by different types of disasters. Natural disasters, such as earthquakes and hurricanes, often lead to abrupt healthcare disruptions, causing treatment interruptions, medication shortages, and acute exacerbations of chronic conditions like hypertension, diabetes, and cardiovascular disease. For example, studies from Japan and Taiwan reported spikes in hypertension, stroke, and myocardial infarction following earthquakes, linked to stress and disrupted continuity of care [37,38,39,40,41,42]. In contrast, complex emergencies involving conflicts and displacement, such as in Syria and Ukraine, exert prolonged pressures on already fragile health systems, undermining chronic care infrastructure, displacing vulnerable populations, and creating long-term medicine shortages [43,44]. Our findings suggest that EMTs responding to both natural and human-made disasters must anticipate and adapt to the specific NCD-related risks associated with the nature and timeline of the emergency. Customizing EMT responses based on the disaster type and the health needs of affected populations could significantly enhance the quality and effectiveness of care delivered in crisis settings. Tailoring EMTs’ response to reflect the specific epidemiological burden and infrastructure challenges of the affected area allows for more targeted and responsive interventions.

Despite the recognition of EMTs as key actors in humanitarian response, the findings of this review highlighted critical gaps across multiple intervention areas. In the preoperational phase of EMTs’ deployment, it should be considered that the type of health problems during disasters might significantly change from the beginning to the late phase of the EMTs’ response. In the immediate aftermath (24–48 h), the team can expect to receive patients with problems directly related to the event, such as traumatic injuries and acute mental health issues [17,23], while after 15 days the patients in need of assistance will have mostly routine healthcare and follow-up issues [17,18,23]. In addition, the whole time needed to become operational can be extremely variable depending on the disaster’s type and location, and on the coordination and registration procedures set up in the affected country [23]. These considerations suggest that EMTs who become operational two weeks after the disaster’s onset have a higher probability of being involved in chronic health needs management. Furthermore, the fact that non-disaster-related events increased as time passed after the disaster [16,18,27,30] posed relevant implications concerning the EMTs’ length of stay in the affected areas. The provision of prolonged EMT services could ensure the continuity of care to patients with chronic NCDs until the reestablishment of local health service delivery [17,27]. Given these points, the EMT staff composition should be constituted and adapted based not only on the nature of the emergency but also taking into account the different phases of the response. The constitution of an EMT staff trained to manage chronic health needs could be obtained through the adoption of multidisciplinary teams with an expanded range of skills and competences [16,26] or through the implementation of NCDs specialized cells able to integrate into EMT types 1, 2, and 3 [24]. Among the intervention areas identified in this review, ensuring a consistent supply of essential medications for patients with NCDs emerged as a critical challenge across all phases of EMT deployment. In the pre-deployment phase, logistical planning is often hindered by limited access to reliable epidemiological data on the chronic disease burden in the affected area, making it difficult to anticipate specific pharmaceutical needs. During the acute response phase, EMTs must operate under constrained supply chain conditions, often in environments where infrastructure is damaged, customs procedures are delayed, and cold chain requirements for medications like insulin are difficult to maintain. Our findings suggest that enhancing EMTs’ medication inventories with a range of essential chronic diseases (e.g., antidiabetics, antihypertensives, bronchodilators, and analgesics) is essential [21,26,27,29]. Moreover, the provision of medical devices to monitor blood and urine glucose levels [27], the utilization of surgical kits to manage diabetic wounds [27], and to guarantee specialized care, such as ophthalmic care, were indicated as useful tools to address the health needs of vulnerable populations. Furthermore, the integration of X-ray [21] and ultrasonography [32] tools in the standard clinical equipment of mobile teams could represent a useful strategy to increase the EMT diagnostic capacity. These findings showed alignment with the recent scientific literature, which underlined the importance of establishing strategic plans aimed at guaranteeing the provision of NCD medical supplies [8]. In this context, a list based on the WHO Interagency emergency health kits [45], on the Package of Essential Non-Communicable Diseases (PEN) [14], and on the national essential drugs list was recently suggested to avoid NCD treatment interruptions in case of disaster response [13,14].

Finally, the implementation of data collection and reporting systems emerged as a key point to better define EMTs’ activities related to NCDs management [19,21], to guarantee the continuity of care [16,20,24], and to facilitate the reintegration of patients into the local health system [29]. Moreover, a more precise identification of NCDs and mental health needs in the WHO EMTs Minimum Data Set (MDS) should be considered, in order to allow a more precise identification of EMTs’ clinical activities [21,22]. These considerations confirm the results of previous authors who emphasized the need for standardized data collection methods in humanitarian crises [46].

Continuity of care beyond the acute crisis phase remains one of the most significant challenges in NCD management during disasters. Individuals with chronic conditions often require sustained treatment and follow-up, yet the short-term nature of EMT deployments poses a risk of treatment discontinuity. To address this, EMTs should prioritize early and structured collaboration with local healthcare providers to enable a seamless transition of care. The integration and collaboration with community health workers, community-based organizations, and local health facilities emerged as a key element to ensure the continuous treatment of patients with chronic illnesses [25,29,30,31,32] and to guarantee the sustainability of EMTs’ interventions [16,26]. Working in integration and not in overlap was reported as a fundamental need useful to support an effective training of local health workers and to promote health education of the affected population [16,18,21,26]. These findings confirmed the results of previous studies [31] and showed alignment with WHO guidelines for emergency medical teams [2]. Moreover, working closely not only with local healthcare providers but also with Non-Governmental Organizations (NGOs) was suggested as essential for maintaining continuity of care [30]. This finding was supported by recent publications that highlighted the important role of NGOs in supporting countries during emergencies and disasters [47,48].

Another often overlooked yet critical dimension of NCD management in disasters is the role of the family. In disaster settings, family members often take on essential caregiving responsibilities such as medication administration, transport to health facilities, wound care, and adherence monitoring [33]. Studies have shown that family members often act as intermediaries with health providers, advocates for needed services, and sources of vital health history, particularly when medical records are inaccessible or destroyed [34]. Despite this, the role of family caregivers is rarely acknowledged in disaster response planning or EMT deployment protocols. Future guidelines for EMTs should therefore consider the integration of family-focused strategies as a key component of comprehensive NCD response planning.

Taken together, our findings suggested that personalizing care for individuals with chronic NCDs in disaster contexts requires both structural and operational changes in EMT deployment. In this regard, several authors highlighted the need to broaden the scope of EMTs’ activities to include not only acute care but also routine clinical services and specific assistance for chronic NCDs [16,18,19,22,28,29,30,31,32]. Addressing this need involves integrating long-term health planning into emergency response operations and adapting EMTs’ roles to meet the ongoing and often complex needs of patients with chronic conditions. Integrating strategies for personalized care, such as individualized treatment plans, proactive identification of high-risk patients, continuity of pre-disaster medication regimens, and the deployment of relevant clinical specialists (e.g., internal medicine specialists, cardiologists, or mental health professionals) can significantly improve health outcomes. This person-centered approach is especially critical for individuals with chronic conditions, whose care requires ongoing monitoring and access to appropriate pharmacological therapies.

To translate these insights into practice, future EMTs’ deployment models could greatly benefit from dedicated training and simulation exercises to prepare teams for evolving challenges of chronic disease and mental healthcare. Based on the findings of this review, we respectfully propose two key recommendations that may support WHO’s efforts to strengthen preparedness and continuity of care in emergency settings. First, WHO might consider the potential value of expanding the EMTs Classification and Minimum Standards (“Blue Book”) to incorporate more detailed, context-sensitive guidance on the management of chronic NCDs. Tailoring such guidance to the specific types of EMTs and the distinct phases of deployment could promote greater adaptability and improve overall effectiveness in the field. Second, incorporating a more detailed classification of chronic conditions into the EMT Minimum Data Set (MDS) could significantly enhance situational awareness, improve continuity of care, and support more effective long-term response planning. In parallel, fostering early coordination between surge teams and local health facilities, alongside the prepositioning of essential medications and supplies for NCDs in disaster-prone areas, may further strengthen the integration and sustainability of chronic care within emergency response operations. These measures are closely aligned with WHO’s ongoing commitment to people-centered, equitable healthcare in crisis settings and may contribute to building more resilient and responsive health systems globally.

### Limitations

The main limitations of this scoping review concerned the limited number of papers included, which was attributable to the scarcity of publications focused on EMTs and NCDs management. Moreover, the retrieved literature was highly heterogeneous in terms of study design, scope, and level of detail, which limited the comparability of findings and made it difficult to draw generalizable conclusions. Not all EMTs reported in the studies were formally classified under the WHO EMTs framework, and disaster types varied widely, including both natural and human-made events. This variability limited the possibility of fully standardizing EMT classifications and disaster contexts across studies. We addressed these constraints by adopting a flexible but systematic extraction approach and acknowledged these challenges in our analysis and interpretation. Further research is needed to deeply understand EMTs and NCDs management, in order to better define standard operating procedures that address this issue during disaster responses. Both quantitative and qualitative studies could be useful for this purpose. The former would allow us to increase the amount of clinical data available; the latter could be relevant to outline possible NCD management strategies arising from interviews and focus group discussions with actors, experts in this field.

## 5. Conclusions

To the best of our knowledge, this is the first systematic review focused on EMTs and NCDs management. Although NCDs are largely recognized as an increasing global health challenge during disasters, the low number of studies that met the inclusion criteria demonstrated that the topic is still relatively unexplored. The findings emerging from this study showed that NCDs significantly impact disaster response in different settings, underlining the need to implement a range of EMT activities to guarantee assistance for chronic health needs. In view of strengthening the ability of health systems to cope with the NCDs burden, the EMTs’ initiatives should be considered as a bridge between the support provided during the acute phase of an emergency and the continuation of care ensured by the system in its early recovery phase.

## Supplementary Materials

The following supporting information can be downloaded at: https://www.mdpi.com/article/doi/s1, Table S1: Population, Concept, and Context (PCC) according to the JBI’s methodological approach to conducting scoping reviews. Table S2: Data collection table.
