# Supplementary material for: Non-Communicable Disease (NCD) Management During Disasters and Humanitarian Emergencies: A Review of the Experiences Reported by Emergency Medical Teams (EMTs)
Source: J Pers Med. 2025 Jun 16;15(6):255. doi: 10.3390/jpm15060255 (PMC12193765; doi:10.3390/jpm15060255)
Supplement: Supplementary file 1 [file jpm-15-00255-s001.zip › Supplementary Table S1_PCC framework.pdf]

Table S1. Research question, frame search and inclusion criteria developed using the PCC elements with 'P' denoting population, 'C', the concept and 'C', the context according to the JBI's methodological approach to conducting scoping reviews.

**RESEARCH QUESTION**		
How did Emergency Medical Teams (EMTs) manage NCDs during disasters and humanitarian emergencies?		
**FRAME SEARCH**		
Pubmed	((((emergency medical team* [MeSH Terms]) OR ET [Title/Abstract])) OR ("Medical team*" [Title/Abstract])) AND ((((((((disaster[MeSH Terms]) OR "Humanitarian emergenc*" [Title/Abstract])) OR (conflict* [Title/Abstract])) OR (flood* [Title/Abstract])) OR (war* [Title/Abstract])) OR (cyclon* [Title/Abstract])) OR (earthquake* [Title/Abstract])) OR (pandem* [Title/Abstract]))) AND ((((non communicable diseas*[MeSH Terms]) OR NCD*[Title/Abstract])) OR ("chronic disease*"[Title/Abstract])) OR "chronic health need*"[Title/Abstract]))	
EBSCO (CINAHL,MEDLINE, PSycinfo)	SU emergency medical team AND SU (non-communicable diseases or ncds or ncd or noncommunicable diseases) AND SU disaster AND SU (humanitarian aid or humanitarian assistance or humanitarian relief or humanitarian action or emergency)	
SCOPUS	(TITLE-ABS-KEY (emergency AND medical AND teams) OR TITLE-ABS-KEY (emts) AND TITLE-ABS-KEY (ncds) OR TITLE-ABS-KEY (non AND communicable AND diseases) OR TITLE-ABS-KEY (chronic AND diseases) AND TITLE-ABS-KEY (disasters) OR TITLE-ABS-KEY (humanitarian AND aid))	
**SELECTION CRITERIA**		
	Inclusion	Exclusion
Population	Emergency Medical Teams (EMTs), including both WHO classified and not-WHO classified EMTs	Studies not including Emergency Medical Teams

Concept	NCDs management (diagnosis, therapy, specialised staff recruitment and deployment, specialised equipment, specialised system)	Studies not reporting information related to NCDs management during the EMTs deployment
Context	Disasters or humanitarian emergencies settings (earthquake, floods, cyclones, war, conflicts)	Studies not concerning disasters or humanitarian emergency settings
