# Supplementary material for: Non-Communicable Disease (NCD) Management During Disasters and Humanitarian Emergencies: A Review of the Experiences Reported by Emergency Medical Teams (EMTs)
Source: J Pers Med. 2025 Jun 16;15(6):255. doi: 10.3390/jpm15060255 (PMC12193765; doi:10.3390/jpm15060255)
Supplement: Supplementary file 1 [file jpm-15-00255-s001.zip › Supplementary Table S2_Data Collection TABLE.pdf]

Table S2. Data collection table**.**

MANUSCRIPT GENERAL INFORMATION				DISASTER		EMERGENCY MEDICAL TEAMS (EMTS)									NON COMMUNICABLE DISEASES (NCDs)			MAIN FINDINGS			
**1st Author/Year of publication**	**Title**	**Type of study**	**Aim**	**Disaster**	**Country/Year of occurrence**	**EMT type**	**EMT sending institution**	**EMT location**	**EMT staff**	**EMT capacity**	**Time of deployment/Operational time**	**Length of stay**	**Equipment (related to NCDs)**	**EMT training /background**	**NCDs of interest**	**Patients treatment**	**Other actions taken**	**Results**	**Challenges**	**Suggestions**	**Limitations of the study**
Dulacha D/2022	Use of mobile medical teams to fill critical gaps in health service delivery in complex humanitarian settings, 2017-2020: a case study of South Sudan	Descriptive analysis	Analyse the key achievements of emergency mobile medical teams (eMMT) in disaster settings South Sudan	Conflicts, floods, famine, disease outbreaks	South-Sudan/2017-2020	Emergency mobile medical team (eMMT) -> EMTs type 1 mobile	WHO South Sudan	South-Sud(conflict-affected locations such as Kajo-Keji and Tambura from 2017 to 2020, and flood-affected locations such as Pibor, Akobo and Mayom in 2019 and 2020)	Epidemiologists, clinicians or doctors, nurses, laboratory specialists, nutritionists, health promotion experts, and public health officers or water, sanitation, and hygiene (WASH) experts	Not specified	24-48 hours after disasters	Not specified	*Materials and tools:* -> they are dependent on the intervention planned -> they include: -Ministry of Health (MoH) outpatient registers (for outbreaks)- referral forms -information, education and communication (IEC) materials - emergency health kits -laboratory sample collection kits - water sample collection and testing kits, -first aid supplies<br><br>*Operation and logistics support:* - WHO field teams who support securing accommodations and local transportation -United Nations Humanitarian Air Services (UNHAS) support transportation from the duty station to response sites<br><br>*Financing:* interventions are mostly supported through emergency donor-funded projects focused on providing short-term humanitarian aid during acute emergencies	Not specified	Chronic conditions not specified	*Health services implementation:* - preventive services (routine vaccination, antenatal care, health education and promotion) -curative services: (i.e. outpatient consultation, nursing care, minor surgeries, and referral for severe cases -nutrition services	*Implementation of vaccination activities*<br><br>*Alert verification and outbreak investigation*	-320,988 consultations conducted -148,726 under-5-year-old children vaccinated -355,790 individuals vaccinated with oral cholera vaccines in 2018 - 63,280 individuals (round 1) and 64,137 people (round 2) vaccinated with oral cholera vaccines in 2020 -550 healthcare workers were trained by eMMT(mainly on Ebola virus disease and COVID-19 disease) in 2019 and 2020	- Unpredictable security situations, -Poor road and transportation networks -Absence of strategies to ensure the continued provision of vaccination services and the management of the chronic conditions initiated during the mobile outreach -The lack of predictability of the visit by the mobile teams may negatively affect the community´s uptake of the services -Weak public health surveillance - Poor mobile network coverage	-Including psychosocial, gender-based violence services and mental health care - capacity building and collaborating with community health workers, community based organisations, and local health facilities - strengthening the referral network between mobile outreach sites and functional health facilities - strengthening coordination with local authorities, partners and security apparatus -adoption of digital health for data capture, reporting, and offering of remote health care services, including training health workers - sharing the data from the mobile clinics with the health facilities serving the catchment populations	- Chronic conditions managed during the mobile outreach are not deeply specified in this report

MANUSCRIPT GENERAL INFORMATION				DISASTER		EMERGENCY MEDICAL TEAMS (EMTS)									NON COMMUNICABLE DISEASES (NCDs)			MAIN FINDINGS			
**1st Author/Year of publication**	**Title**	**Type of study**	**Aim**	**Disaster**	**Country/Year of occurrence**	**EMT type**	**EMT sending institution**	**EMT location**	**EMT staff**	**EMT capacity**	**Time of deployment/Operational time**	**Length of stay**	**Equipment (related to NCDs)**	**EMT training /background**	**NCDs of interest**	**Patients treatment**	**Other actions taken**	**Results**	**Challenges**	**Suggestions**	**Limitations of the study**
Yumiya Yu/2022	Prevalence of Mental Health Problems among Patients Treated by Emergency Medical Teams: Findings from J-SPEED Data Regarding the West Japan Heavy Rain 2018	Descriptive analysis	To examine how mental health needs are accounted for in the overall picture of disaster relief and how they change over time	West Japan Heavy West Japan Heavy Rain	Japan/2018<br>(8 July - 11 September 2018)	(Type 1 and 2) Different EMTs: the Disaster Medical Assistance Teams (DMATs), the Japan Medical Association Team (JMAT), and the Japan Red Cross Medical Assistance Team (JRC), Disaster Psychiatric Assistance Teams (DPATs)<br><br>-> to provide not only physical, but also psychological care, such as psychological first aid (PFA) and psychiatric medical treatment during the acute phase	NGO: Japan Medical Association ->JMAT<br><br>Government: ->DMATS -> DPAT?<br><br>Independent organisation: Japan Red Cross -> JRC	West Japan	Not specified	Not specified	Not specified	41 days	Not specified	Psychological first aid (PFA)<br><br>Psychiatric support	Mental health's problems (Disaster stress-related symptoms included insomnia, headache, dizziness, loss of appetite, gastric pain, and constipation, and mental care needs included suicide attempts, problem behaviors and attitudes, and restlessness)	Not specified	Not specified	- A total of 372 (10.3%) mental health consultations were recorded out of 3617 total consultations<br>- Among these, 205 (9.9%) were for patients aged 9–74 years, and 161 (11.5%) were for people aged ≥75 years<br>- Of the 372 consultations recorded, the majority (83.1%) occurred (mainly from day 1 to day 7<br><br>- During the first 2 weeks, the proportion of patients aged 9–74 years was larger than that of patients aged ≥75 years<br>- patients aged ≥75 years were more likely to visit later<br><br>-> the daily percentage of mental health problems significantly decreased from the beginning of the	Not specified	-It is important to follow a recovery approach based on the extent of damage, phased time periods, and psychological resilience<br><br>-A sufficient number of EMTs who can provide MHPSS, such as DPATs, needs to be ensured<br><br>- EMTs should be trained in mental health care, such as PFA ->EMTs screen for psychiatric symptoms, identify high-risk patients through primary care, and refer them to mental health professionals<br><br>-more specialised teams with psychological experts for children<br><br>- In the case of an exit strategy, it is important to ensure a continuous service provision system for patients<br><br>->the percentage of patients with mental health problems may be used as an indicator to detect phase changes	- Mental health issues could be underdetected (it is more difficult to detect mental health issues than physical illnesses)<br><br>- Lack of data concerning childrens' mental health (underreporting seems to be particularly pronounced in children who are unable to communicate their symptoms)<br><br>- Data form only one disaster

MANUSCRIPT GENERAL INFORMATION				DISASTER		EMERGENCY MEDICAL TEAMS (EMTS)									NON COMMUNICABLE DISEASES (NCDs)			MAIN FINDINGS			
**1st Author/Year of publication**	**Title**	**Type of study**	**Aim**	**Disaster**	**Country/Year of occurrence**	**EMT type**	**EMT sending institution**	**EMT location**	**EMT staff**	**EMT capacity**	**Time of deployment/Operational time**	**Length of stay**	**Equipment (related to NCDs)**	**EMT training /background**	**NCDs of interest**	**Patients treatment**	**Other actions taken**	**Results**	**Challenges**	**Suggestions**	**Limitations of the study**
																		response period to the 19th response day, and then gradually increased (V-shaped pattern)			
Sacchetto D/2022	Italian Field Hospital Experience in Mozambique: Report of Ordinary Activities in an Extraordinary Context	Descriptive analysis	To report the mission of the EMT2-ITA in Mozambique, raising interesting points of discussion regarding the impact of timing on the mission outcomes, the operational and clinical activities in the field hospital, and the great importance to integrate local staff into the team	Cyclone Idai	Mozambique /2019 (March 15)	Emergency Medical Team Type 2 – Italy Regione Piemonte (EMT2-ITA)	Italian Government	Beira (courtyard of the Central hospital of Beira)	58 health care professionals (29 medical doctors, including two team leaders and one deputy team leader; 27 nurses; one x-ray technician; and one midwife) and 19 operation support personnel (one engineer; four electricians; two water, sanitation, and hygiene (WASH) experts; and 12 logisticians	24-hour operativity, to provide seven major/fifteen minor surgical operations per day, to manage 100 out-patients and 20 in-patients per day, totally self-sufficient from a logistical point of view	Two weeks after the disaster	27 days	Not specified	Not specified	Cardiovascular Diseases (4.2%)<br><br>Neurological diseases (5.2%)<br><br>Respiratory diseases (7.1%)	Not specified	Surgical procedures, mainly elective (53.2%)	1,121 patients were treated<br><br>1,183 triage admissions (several representations)<br><br>Only few patients with specific disaster-related injuries -> many patients coming to the field hospital for routine medical care	Under-utilisation of the in-patient wards<br>No patients presented at night as transportation became much more difficult after dark -> from 12 pm to 8 am on-call availability was organised	EMTs must be ready to cope not only with daily emergencies but also with routine activities during their missions<br><br>The importance of complementary role of the EMT2-ITA for the local central hospital<br><br>The importance of integration of the local staff in the team composition during the rotation of the personnel of the EMT2-ITA<br>-> to limit the number of professionals coming from Italy<br>-> to train the local staff<br><br>The main role of an EMT becoming operative several days after a disaster event is the full commitment for elective activities to support and maintain the ordinary health care	

MANUSCRIPT GENERAL INFORMATION				DISASTER		EMERGENCY MEDICAL TEAMS (EMTS)									NON COMMUNICABLE DISEASES (NCDs)			MAIN FINDINGS			
**1st Author/Year of publication**	**Title**	**Type of study**	**Aim**	**Disaster**	**Country/Year of occurrence**	**EMT type**	**EMT sending institution**	**EMT location**	**EMT staff**	**EMT capacity**	**Time of deployment/Operational time**	**Length of stay**	**Equipment (related to NCDs)**	**EMT training/background**	**NCDs of interest**	**Patients treatment**	**Other actions taken**	**Results**	**Challenges**	**Suggestions**	**Limitations of the study**
																				capacity of the affected country	
Odgerel Chimed-Ochir/2022	Emergency Medical Teams' Responses during the West Japan Heavy Rain 2018: J-SPEED Data Analysis	Descriptive epidemiology study	To better understand the health problems during floods and heavy rain disasters	West Japan Heavy Rain	Japan/8 July - 11 September 2018	Not specified (85 EMTs)	Not specified	Hiroshima, Okayama, and Ehime	Not specified	Not specified	Not specified	(65 days) From 8 July to 11 September 2018	Not specified	Not specified	Hypertension, deep vein thrombosis, disaster stress related symptoms	Not specified	Not specified	Out of 3,617 consultations recorded, skin disease accounted for the highest percentage (627; 17.3%) followed by wounds (518; 14.3%), non-disaster-related events (383; 10.6%), disaster stress-related symptoms (361; 10.0%), conjunctivitis (228; 6.3%), acute respiratory infections ([ARI] 195; 5.4%), and heatstroke (183; 5.1%)<br><br>Hypertension (5.1%), deep vein thrombosis (0.8%), urgent need for psychological support (0.3%), and interrupted essential medications (4.6%), needs of transfer (0.7%), and non-disaster-related		It is critical to ensure that patients with chronic conditions are monitored and their medication is maintained during floods<br><br>Non-disaster-related events increased as time passed after the disaster. During the response, the transition of this characteristic was used as an indicator to guide and support decision to demobilise the EMTs.<br><br>Data collection is crucial (the system J-SPEED is a new standard data reporting system among Japanese EMTs)	J-SPEED reporting was a new system:<br>-> lack of pre-training<br>-> lack of understanding<br><br>J-SPEED is mainly used to collect data from EMTs rather than local health facilities<br><br>-> underestimation of health diseases<br><br>-> data collected were "dirty" or missing

MANUSCRIPT GENERAL INFORMATION				DISASTER		EMERGENCY MEDICAL TEAMS (EMTS)									NON COMMUNICABLE DISEASES (NCDs)			MAIN FINDINGS			
**1st Author/Year of publication**	**Title**	**Type of study**	**Aim**	**Disaster**	**Country/Year of occurrence**	**EMT type**	**EMT sending institution**	**EMT location**	**EMT staff**	**EMT capacity**	**Time of deployment/Operational time**	**Length of stay**	**Equipment (related to NCDs)**	**EMT training /background**	**NCDs of interest**	**Patients treatment**	**Other actions taken**	**Results**	**Challenges**	**Suggestions**	**Limitations of the study**
																		events(10.1%) were highest among elderly patients (over 75 years old)			
McMaster D/2022	Designing a Mobile Eye Hospital to Support Health Systems in Resource-Scarce Environments	Design plan study (discussion paper?)	To proposes a design plan for a mobile eye hospital to support health systems between the initial emergency response and recovery of health infrastructure in resource-scarce environments of low- and middle-income countries.	Not specified	Not applicable	EMT Specialized on eye diseases (a mobile eye hospital)	Not applicable	Not applicable	Ophthalmologists, ophthalmic assistants, nurses, anesthesiologist	Not specified	Not specified	Not specified	An expandable shipping contain and a rapid assembly field tent<br><br>Examination equipment shoul include visual acuity charts, a portable slit lamp indirect ophthalmoscope tonometer, fundu lenses, compact A/B ultrasound scanner, autorefractor, sphygmomanomter, and expendable supplies such as eye drops and reagent for estimating urine sugar<br><br>For transportatio a truck capable o delivering a standard 20-ft shipping contain to the target location is the minimum requirement<br><br>Operating room should be equipped with essential emergency equipment, including airway and breathing equipment, intravenous and intraosseous access, and essential medicines including intravenous fluid and antibiotics<br><br>A satellite Interne connection and a way of storing electronic medic data	Ophthalmologists must be trained in basic and advanced life support with guidance from intensive care specialists and anesthetists to identify warning signs and understand acute management of emergencies, including resuscitation	Eye diseases (including diseases caused by NCDs)	Medical evaluation<br><br>Surgery treatment<br><br>Laser treatment				There is a need to provide a semi-permanent service between the initial emergency response and the reestablishment of local health service delivery.<br><br>EMT self-sufficient as possible, including provision of food, water, security, and medical waste disposal	

MANUSCRIPT GENERAL INFORMATION				DISASTER		EMERGENCY MEDICAL TEAMS (EMTS)									NON COMMUNICABLE DISEASES (NCDs)			MAIN FINDINGS			
**1st Author/Year of publication**	**Title**	**Type of study**	**Aim**	**Disaster**	**Country/Year of occurrence**	**EMT type**	**EMT sending institution**	**EMT location**	**EMT staff**	**EMT capacity**	**Time of deployment/Operational time**	**Length of stay**	**Equipment (related to NCDs)**	**EMT training /background**	**NCDs of interest**	**Patients treatment**	**Other actions taken**	**Results**	**Challenges**	**Suggestions**	**Limitations of the study**
Hirokazu Tachikawa /2022	Mental health needs associated with COVID-19 on the diamond princess cruise ship: A case series recorded by the disaster psychiatric assistance team	Case series	To Assess the clinical characteristics of patients with acute mental health needs on a quarantined ship and recommend evidence-based measures for disaster mitigation	COVID-19Pandemic	Japan, Diamond Princess cruise ship/February 2020	Disaster Psychiatric Assistance Team (DPAT)	MoH Japan	Diamond Princess cruise ship	Fifty-five members of 12 DPAT groups					Psychologists/Psychiatrists	Mental health issues	Psychological advice and Psychiatric assistance	n	Mental health issues including disaster stress related symptoms were as frequent as physical health events associated with COVID-19. The most significant mental health issue was anxiety, as an acute psychological reaction to the quarantine situation. Women and crews most frequently needed mental health support. Mental health improved in most clients after brief counseling. Although several passengers experienced suicidal ideation, there were no cases of actual suicide attempts during the quarantine period.		Disaster mental health services such as DPATs is essential companions to medical services for the maintenance of public health during crisis situations. Mental health issues experienced onboard the Diamond Princess cruise ship shed light on psychological responses to disease outbreaks on quarantined ship<br>-> The mental health version of the J-SPEED/WHO-MDS	First, the study cases were limited to a group of individuals who independently requested health support. Therefore, our findings may not be generalizable to all passengers and crew on the ship. Second, samples were collected from the J-SPEED database, which was used by EMTs during the mission, and some data may be missing or inaccurate. Third, data regarding the medical support provided by the DMAT and JMAT were only collected during the middle of the activity period. Fourth, more women than men were analyzed, since counseling was requested more frequently by women than men. Fifth, each health event was recorded as an individual response. Thus, the number of health

MANUSCRIPT GENERAL INFORMATION				DISASTER		EMERGENCY MEDICAL TEAMS (EMTS)									NON COMMUNICABLE DISEASES (NCDs)			MAIN FINDINGS			
**1st Author/Year of publication**	**Title**	**Type of study**	**Aim**	**Disaster**	**Country/Year of occurrence**	**EMT type**	**EMT sending institution**	**EMT location**	**EMT staff**	**EMT capacity**	**Time of deployment/Operational time**	**Length of stay**	**Equipment (related to NCDs)**	**EMT training /background**	**NCDs of interest**	**Patients treatment**	**Other actions taken**	**Results**	**Challenges**	**Suggestions**	**Limitations of the study**
																					events did not reflect the number of individuals who requested assistance .
Ning-Ping Foo/2021	Establishment of disaster medical assistance team standards and evaluation of the teams' disaster preparedness: An experience from Taiwan	Two phases. Phase I was a Delphi study conducted in 2019 with 26 experts who were invited to establish Taiwan's DMAT standards by modifying the World Health Organization Emergency Medical Team (WHO EMT) type I fixed standards. Phase II was a cross-sectional study conducted in 2020. A questionnaire was used to evaluate the disaster preparedness of DMATs by standards set in phase I	To develop localised DMAT (dIsaster Medical Assistance Teams) standards for Taiwan by referring to EMT type I standards	Not related to a specific disaster	Not related to a specific disaster	Type 1 fixed	Taiwan Government, but also private institutions		Suggestions : 1:2:2 ratio of physicians:nurses:logisticians	Suggestion : 100 outpatient per day/s 24h			Ultrasound services were added	EMergency physicians (selection bias?) Logistics personnel were expected to have an emergency medical technician license	Emergency chronic disease care					EMT Type 1 fixed need more: unified government announcement standards, legal protection, and adequate financial support.	Selection bias for the participants a phase III study with on-site formal interviews in the future could resolve this problem
Ladeira LM/2021	PT EMT – Portuguese Emergency Medical Team Type 1 Relief Mission in Mozambique	Descriptive analysis	To report the mission of the PT EMT type 1 in Mozambique	Cyclone Idai	Mozambique/2019	EMT Type 1 fixed	Portuguese National Institute of Medical Emergency (INEM; Lisboa, Portugal), a government agency under the umbrella of the Portugal Ministry of Health	Beira	Doctors of different specialties (ie, intensive care, internal medicine, pediatrics, surgery, obstetrics, and infectious diseases); specialized nurses in critically ill patients, or	Two-team rotation (28 elements each)<br><br>The PT EMT infrastructure has several care areas, namely an emergency room, ambulatory ,	15 days (the PT EMT deployed immediately an assessment team of three members who worked with the local emergency manage	30 days (1-30 April 2019)	X-ray very important. - it was used in 27 cases (16.6%), namely in limb trauma (27.5%) and medical situations (72.5% %)<br><br>No further data provided	Not specified	Low back pain headache and gastritis	Analgesic drugs administration<br><br>No further data provided (concerning NCDs)	Training sessions were promoted with local staff	1,662 patients were admitted to PT EMT<br><br>The five most prevalent diagnoses were: 61.49% classified with "code 29" (which	The need of addressing the inter-EMT transport in order to enable a true network between EMTs and local health care structure<br><br>Minimal Data Set	The WHO EMTs Minimum Data Set should consider a more precise identification of non-communicable diseases, as this can be the main activity in some deployments	

MANUSCRIPT GENERAL INFORMATION				DISASTER		EMERGENCY MEDICAL TEAMS (EMTS)									NON COMMUNICABLE DISEASES (NCDs)			MAIN FINDINGS			
**1st Author/Year of publication**	**Title**	**Type of study**	**Aim**	**Disaster**	**Country/Year of occurrence**	**EMT type**	**EMT sending institution**	**EMT location**	**EMT staff**	**EMT capacity**	**Time of deployment/Operational time**	**Length of stay**	**Equipment (related to NCDs)**	**EMT training /background**	**NCDs of interest**	**Patients treatment**	**Other actions taken**	**Results**	**Challenges**	**Suggestions**	**Limitations of the study**
									emergency and disaster; prehospital technicians; psychologists; x-ray technicians; pharmaceuticals; and logistical elements	minor surgery/orthopedics, pediatrics, pharmacy, and x-ray	ment authority to evaluate necessitie ->establishment of the first EMT Coordination Cell to evaluate the needs and to work in collaboration (not in overlap) with local authorities							corresponds to “other unspecified diagnosis”), 9.15% of cases of skin disease, 8.90% of minor injuries, 6.74% of acute respiratory infection, and 3.19% of obstetric/gynecologic complications Among “code 29” the most prevalent of diagnosis were low back pain (11.47%) Analgesic drugs being the most consumed (48.2%). Antibiotics had a reduced consumption (16.1%). , headache (6.37%), and gastritis (5.86%)<br><br>Among patients (88.61% were discharged permanently, 7.83% were identified for follow-up (n = 130), and 3.31% (n = 55) were transferred to the Spanish EMT-2	register: code 29 is insufficient to allow an adequate classification of all the clinical conditions managed	The WHO and the EMT initiative should include a transportation cell<br><br>EMTs should work with local health authorities and Mozambican health professional<br><br>The importance of X-Ray and the use of analgesic drugs could be used to better define EMT’s equipment and pharmacological load	

MANUSCRIPT GENERAL INFORMATION				DISASTER		EMERGENCY MEDICAL TEAMS (EMTS)									NON COMMUNICABLE DISEASES (NCDs)			MAIN FINDINGS			
**1st Author/Year of publication**	**Title**	**Type of study**	**Aim**	**Disaster**	**Country/Year of occurrence**	**EMT type**	**EMT sending institution**	**EMT location**	**EMT staff**	**EMT capacity**	**Time of deployment/Operational time**	**Length of stay**	**Equipment (related to NCDs)**	**EMT training/background**	**NCDs of interest**	**Patients treatment**	**Other actions taken**	**Results**	**Challenges**	**Suggestions**	**Limitations of the study**
Bartolucci A/2020	Decision Support Framework for Deployment of Emergency Medical Teams After Earthquakes	Desk-based study	To enhance disaster managers' literacy and to provide a framework that will assist those responsible for deploying and/or accepting EMTs in making informed decision on deployment of emergency medical teams after an earthquake	Earthquakes	Not specified	EMT type 1,2,3	------------	------------	Not specified	Not specified	Days:<br><br>0/2<br>3/5<br>5/15<br>>15	Not specified	Not specified	Not specified	Not specified "(general care", "non urgent conditions" , "unrelated chronic health conditions")	Not specified	________	5 main time phases in the mobilization of an EMT to becoming operational in the affected area: (1) Request For Assistance (RFA); (2) Loading time; (3) Travel time; (4) Entry time; and (5) Set up/tasking time<br><br>The RFA time considers the time necessary for the affected country to make the assessment of needs, determining whether the international assistance is required, and terminates with the formal request of international assistance<br><br>The Loading time identifies the time from the RFA to the departure of the team to the	Difficulties in standardize the time of deployment<br><br>Unpredictability of idsasters, logistical complications, local protocols procedures that can affect the METs registration and location assignments, type of EMTs	To test and validate the framework, for instance in training courses and programs such as the TEAMS project, the Emergency Medical Team Coordination Cell (EMTCC) Training Course, and United Nations Disaster Assessment and Coordination (UNDAC) Induction Course<br><br>To use the framework as a basis for the creation of a Decision Support System (DSS) that can could help the WHO Coordination Centre, host governments, and decision makers to decide whether to deploy or accept a team	Simplification of the deployment process (the framework does not consider specific parameter such as country,type of EMTs and specific context related logistical problems)

MANUSCRIPT GENERAL INFORMATION				DISASTER		EMERGENCY MEDICAL TEAMS (EMTS)									NON COMMUNICABLE DISEASES (NCDs)			MAIN FINDINGS			
**1st Author/Year of publication**	**Title**	**Type of study**	**Aim**	**Disaster**	**Country/Year of occurrence**	**EMT type**	**EMT sending institution**	**EMT location**	**EMT staff**	**EMT capacity**	**Time of deployment/Operational time**	**Length of stay**	**Equipment (related to NCDs)**	**EMT training /background**	**NCDs of interest**	**Patients treatment**	**Other actions taken**	**Results**	**Challenges**	**Suggestions**	**Limitations of the study**
																		affected area. It considers the time the assisting nations need to load all the assets, contact all the staff involved, and the shipping of all the assets from the airport. For International EMTs, WHO and International Federation of Red Cross and Red Crescent Societies (IFRC) reports suggest that the loading time should be within 12 h for all the teams.<br><br>The Travel time considers the time necessary to reach the affected nation. The amount of the hours for the framework strongly depend on the location of the disaster<br><br>The Entry time considers the time needed			

MANUSCRIPT GENERAL INFORMATION				DISASTER		EMERGENCY MEDICAL TEAMS (EMTS)									NON COMMUNICABLE DISEASES (NCDs)			MAIN FINDINGS			
**1st Author/Year of publication**	**Title**	**Type of study**	**Aim**	**Disaster**	**Country/Year of occurrence**	**EMT type**	**EMT sending institution**	**EMT location**	**EMT staff**	**EMT capacity**	**Time of deployment/Operational time**	**Length of stay**	**Equipment (related to NCDs)**	**EMT training /background**	**NCDs of interest**	**Patients treatment**	**Other actions taken**	**Results**	**Challenges**	**Suggestions**	**Limitations of the study**
																		to the Reception and Departure Centre (RDC) procedures of registration and location assignment.<br><br>The Set-up time is the time from the arrival in the designated area to the complete set up of the team. According to the WHO, EMTs type 1 should be available to arrive in the fastest possible time, ideally within 24-48 h, and be considered light and portable while Type 3 EMTs are unlikely to be operational in the field for at least 5-7 d<br><br>In the immediate aftermath of the event (24-48 h), the team can expect to receive 8 of 10 patients with problems directly related to			

MANUSCRIPT GENERAL INFORMATION				DISASTER		EMERGENCY MEDICAL TEAMS (EMTS)									NON COMMUNICABLE DISEASES (NCDs)			MAIN FINDINGS			
**1st Author/Year of publication**	**Title**	**Type of study**	**Aim**	**Disaster**	**Country/Year of occurrence**	**EMT type**	**EMT sending institution**	**EMT location**	**EMT staff**	**EMT capacity**	**Time of deployment/Operational time**	**Length of stay**	**Equipment (related to NCDs)**	**EMT training /background**	**NCDs of interest**	**Patients treatment**	**Other actions taken**	**Results**	**Challenges**	**Suggestions**	**Limitations of the study**
																		the earthquake, while, after 15 d, the patients will have mostly routine health-care issues. Patients with follow-up needs will start to present from 3 to 15 d			

MANUSCRIPT GENERAL INFORMATION				DISASTER		EMERGENCY MEDICAL TEAMS (EMTS)									NON COMMUNICABLE DISEASES (NCDs)			MAIN FINDINGS			
**1st Author/Year of publication**	**Title**	**Type of study**	**Aim**	**Disaster**	**Country/Year of occurrence**	**EMT type**	**EMT sending institution**	**EMT location**	**EMT staff**	**EMT capacity**	**Time of deployment/Operational time**	**Length of stay**	**Equipment (related to NCDs)**	**EMT training /background**	**NCDs of interest**	**Patients treatment**	**Other actions taken**	**Results**	**Challenges**	**Suggestions**	**Limitations of the study**
McMaster D/2020	Integrating specialist ophthalmic services into emergency medical teams	Discussion paper	To describe the importance of increasing specialist ophthalmic services within emergency medical teams.	Conflicts (not specified), Earthquakes, infectious diseases outbreaks	Conflicts (Timor-Leste and others not specified), Earthquakes (Japan in 2011 and Nepal in 2015)., Ebola (West Africa, 1013-16) and measles outbreaks (Pacific region, 2019)	EMT type 1, 2,3	Not specified	Not specified	Ophthalmologists and ophthalmic personnel<br><br>Specialist ophthalmology units Rapid response teams capable of integrating into type 1, 2 and 3 medical emergency teams; augment local eye camps and mobile eye units providing treatment of acute and chronic conditions<br><br>Additional specialist Within type 2 or 3 medical emergency teams and/or a local hospital member	Not specified	Not specified	Not specified	Mobile eye units – ranging from well-equipped four-wheel drive utility vehicles to large specialist lorries with onboard operating theaters Mobile phone applications to accurately record visual acuity and capture images of the retina. In addition, measurement of intraocular pressure<br><br>Telemedicine<br><br>Mobile phone applications to accurately record visual acuity and capture images of the retina	A minimum proportion of emergency medical teams personnel to be trained to diagnose and manage several acute eye conditions using essential ophthalmic equipment and consumables	Chronic eye diseases such as cataract and glaucoma	Not specified	Not specified	------------	--------------	A minimum proportion of emergency medical teams personnel to be trained to diagnose and manage several acute eye conditions using essential ophthalmic equipment and consumables to ensure basic eye care<br><br>Use of specialist ophthalmology units able to deploy independently or attach to emergency medical teams<br><br>Integrating specialist ophthalmic services into emergency	----------

MANUSCRIPT GENERAL INFORMATION				DISASTER		EMERGENCY MEDICAL TEAMS (EMTS)									NON COMMUNICABLE DISEASES (NCDs)			MAIN FINDINGS			
**1st Author/Year of publication**	**Title**	**Type of study**	**Aim**	**Disaster**	**Country/Year of occurrence**	**EMT type**	**EMT sending institution**	**EMT location**	**EMT staff**	**EMT capacity**	**Time of deployment/Operational time**	**Length of stay**	**Equipment (related to NCDs)**	**EMT training /background**	**NCDs of interest**	**Patients treatment**	**Other actions taken**	**Results**	**Challenges**	**Suggestions**	**Limitations of the study**
													Measurement of intraocular pressure can now be achieved easily using portable equipment							medical teams may be an effective way of managing ocular injuries, reducing unnecessary sight loss and supporting a transition back to local governance as infrastructure and human resources are rebuilt	
Andrea Dunne-SOsa/2019	The Hidden Wounds of Hurricane Dorian	Field Report	To report the mission of HOPE EMergency Response Team	Hurricane Dorian	Bahamas/ 2019	Emergency Response Team	HOPE . American NGO (non governmental organisation)	Northern Bahamas	Medical professional volunteers		4 days		Insulin needles, hygiene kits		Chronic diseases such as diabetes, hypertension, and cancer	Significant impact on populations with chronic diseases such as diabetes, hypertension, and cancer				Engage professional medical teams to provide primary and chronic healthcare needs in a clinical setting Think about continuity of care, stocking pharmacies adequately, facilitating access to prescription drugs, strengthening cold chain mechanisms, restoring supply chains, and educating patients with chronic illnesses on emergency preparedness. conditions. Project HOPE designs medical teams to work alongside, rather than parallel to, existing health care infrastructure—and for longer than the initial two weeks. This allows patients to	

MANUSCRIPT GENERAL INFORMATION				DISASTER		EMERGENCY MEDICAL TEAMS (EMTS)									NON COMMUNICABLE DISEASES (NCDs)			MAIN FINDINGS			
**1st Author/Year of publication**	**Title**	**Type of study**	**Aim**	**Disaster**	**Country/Year of occurrence**	**EMT type**	**EMT sending institution**	**EMT location**	**EMT staff**	**EMT capacity**	**Time of deployment/Operational time**	**Length of stay**	**Equipment (related to NCDs)**	**EMT training /background**	**NCDs of interest**	**Patients treatment**	**Other actions taken**	**Results**	**Challenges**	**Suggestions**	**Limitations of the study**
																				navigate the difficult period following a disaster and connects them to care services for better management of chronic diseases like diabetes. Most importantly, it lays the foundation for the long-term recovery of a health system that can support them<br>Close coordination with the MoF and local Emergency Management Agency	
van Berlaer G/2019	Clinical Characteristics of the 2013 Haiyan Typhoon Victims Presenting to the Belgian First Aid and Support Team	Cross sectional study	To document the demographics, complaints, comorbidities, diagnoses, diagnosis categories, and management of typhoon victims who sought medical assistance in a field hospital of an international EMT and to formulate recommendations for future relief operations	Typhoon Haiyan	Philippines / 8th November 2013	Belgian First Aid and Support Team (B-FAST) -> level 1 field hospital	Belgian Government	South East region of Tacloban	Volunteer team comprised 5 physicians (1 surgeon, 3 anesthesiologists trained in emergency medicine, and 1 paediatrician); 15 skilled nurses, of whom 1 was of Philippine origin; 1 pharmacist; 4 water and sanitation personnel; and 10 logisticians, interpreters, and communication and information specialists	Enhanced level I medical-surgical field hospital with units for 24/7 triage, ambulatory emergency care, surgery, recovery, and psychosocial support, as well as a small short stay unit	8 days after the Typhoon (16th November 2013) (difficulties in reaching the area with large planes transporting 28 tons of material)	4 days (16-20 November 2013)	Preconfigured interagency emergency health kits (IEHKs)<br><br>Laboratory and imaging tests were unavailable	Volunteer trained nurses and physicians (not other details about training)	Diabetes, hypertension, asthma, mental health disorders<br><br>The proportion of 6%, was lower than the range seen in other reports ->young median age of patients -> the peak presentation of these conditions — secondary to the disruption of the health	Registration on admission and simple triage and rapid treatment (START) were performed by trained nurses and physicians<br><br>Paper World Health Organization (WHO) health cards were completed<br><br>Diagnoses were based on complaints and physical examination, as B-FAST had	In accordance with to the B-FAST exit strategy, after 5 days the compound was handed over to a German nongovernmental organisation for continuation of services until the end of January 2014	1267 field hospital patients were reviewed<br><br>28% of the patients suffered from injury, but most presented with nonsurgical diseases (64%), particularly of respiratory (31%), dermatological (11%), and digestive (8%) origin	Difficulties in reaching the area with large planes transporting 28 tons of material<br><br>The widely used basic IEHK does not contain sufficient medication refills for these patients, so B-FAST provided, by own means, refills of medication and distribution of materials like beta-blockers, inhalers, and urine ketone	Stockpiling of disaster-response equipment and drugs in strategic areas in disaster-prone regions<br><br>The education of the population in order to understand regional risks and how to prepare for and respond to disasters are tools to achieve resilience of a country in case of a catastrophe	The lack of uniform standards to register complaints, clinical features, and diagnoses makes comparison to other datasets challenging<br><br>A number of diagnosis remained tentative, since laboratory and imaging tests were unavailable to confirm the cases suspected of

MANUSCRIPT GENERAL INFORMATION				DISASTER		EMERGENCY MEDICAL TEAMS (EMTS)									NON COMMUNICABLE DISEASES (NCDs)			MAIN FINDINGS			
**1st Author/Year of publication**	**Title**	**Type of study**	**Aim**	**Disaster**	**Country/Year of occurrence**	**EMT type**	**EMT sending institution**	**EMT location**	**EMT staff**	**EMT capacity**	**Time of deployment/Operational time**	**Length of stay**	**Equipment (related to NCDs)**	**EMT training /background**	**NCDs of interest**	**Patients treatment**	**Other actions taken**	**Results**	**Challenges**	**Suggestions**	**Limitations of the study**
									The EMT was supported by members of the German International Search and Rescue Team (ISAR)						care system—generally appears some weeks after the onset of the event<br><br>2% of patients lacked routine medication, mostly for the chronic treatment of comorbidities like diabetes and hypertension, and were therefore at risk for deregulation of their clinical condition	limited access to diagnostic capability at the time<br><br>B-FAST reported daily to the Philippine Department of Health using the Surveillance in Post Extreme Emergencies and Disasters (SPEED) template<br><br>Medical supplies primarily used were from **preconfigured interagency emergency health kits (IEHK) IEHKs.**<br><br>**IEHKs:** they not contain oxygen or antidiabetics, as these supplies come in separate additional kits or have to be added from other supplies The most-used treatment was wound care in general (rinsing, disinfection, local antibiotics and povidone iodine, dressings, sutures or stitching, sulfadiazine), followed by pain relief (paracetamol, nonsteroid		Only 53% presented with disaster-related pathology, and 59% showed signs of infection Patients needed wound care (47%), pain relief (33%), or antibiotics (29%); 9% needed procedures, 8% needed fluid therapy, and 5% needed psychological support<br><br>More than 9% of patients (n = 110) reported 1 or 2 comorbidities, most commonly diabetes (n = 23) or arterial hypertension (n = 14). Patients also reported asthma (n = 11), rheumatic diseases (n = 10), pregnancy (n = 8), epilepsy (n = 4), and neoplasms (n = 3)<br><br>Children under 5 years of age were more at risk for infections and	strips<br>Follow-up or referral		meningitis, pneumonia,<br>tuberculosis, and leptospirosis<br><br>The study covered a limited number of patients form a single region of Philippines

MANUSCRIPT GENERAL INFORMATION				DISASTER		EMERGENCY MEDICAL TEAMS (EMTS)									NON COMMUNICABLE DISEASES (NCDs)			MAIN FINDINGS			
**1st Author/Year of publication**	**Title**	**Type of study**	**Aim**	**Disaster**	**Country/Year of occurrence**	**EMT type**	**EMT sending institution**	**EMT location**	**EMT staff**	**EMT capacity**	**Time of deployment/Operational time**	**Length of stay**	**Equipment (related to NCDs)**	**EMT training /background**	**NCDs of interest**	**Patients treatment**	**Other actions taken**	**Results**	**Challenges**	**Suggestions**	**Limitations of the study**
																al antiinflammatory drugs, opioids in oral and parenteral forms, and anesthetic gels for wounds), the distribution of vitamins and minerals (from the IEHK: zinc, iron, vitamin C), and antibiotics (most patients received narrow-spectrum oral antibiotics, except for the surgical patients, who received intravenous broad spectrum antibiotics)<br><br>Antiparasitic drugs and antimycotics were used for 3% and 2% of patients, respectively<br><br>Respiratory relief was mainly achieved by the use of bronchodilators<br><br>Procedures were performed on 9% of patients (n = 115), including 15 major and 30 minor surgical interventions. Forty-two patients had to be transferred to		injuries)			

MANUSCRIPT GENERAL INFORMATION				DISASTER		EMERGENCY MEDICAL TEAMS (EMTS)									NON COMMUNICABLE DISEASES (NCDs)			MAIN FINDINGS			
**1st Author/Year of publication**	**Title**	**Type of study**	**Aim**	**Disaster**	**Country/Year of occurrence**	**EMT type**	**EMT sending institution**	**EMT location**	**EMT staff**	**EMT capacity**	**Time of deployment/Operational time**	**Length of stay**	**Equipment (related to NCDs)**	**EMT training /background**	**NCDs of interest**	**Patients treatment**	**Other actions taken**	**Results**	**Challenges**	**Suggestions**	**Limitations of the study**
																secondary care hospitals for complex surgery, transfusions, and intensive postoperative care<br><br>Psychological support was delivered to 5% of patients (n = 62), and psychotropic drugs to 41 (3%)					
McDermott KM/2017	Management of Diabetic Surgical Patients in a Deployed Field Hospital: A Model for AcuteNon-Communicable Disease Care in Disaster	Report	The authors use their EMT experiences described in this report as a case study of NCD management in a surgical field hospital and describe the insights gained alongside a framework for future responses to improve the care of diabetic patients in the field	Typhoon Haiyan (Yolanda)	Philippines /8th November 2013	Type 2	Australian government	Tacloban city (220,000 population)	2 consecutive acute trauma surgery teams, 1 internal medicine physician per each team, 1 pharmacist per each team, no nursing staff with a primary specialty of patient inward nursing	40 in patients beds, 1 operating table + 1 extra table on-call 24 hours	6 days after the disaster (16 November 2013)	23 days (from 16 November to 7 December 2013)	Blood glucose monitoring<br><br>Dipstick urinalysis<br><br>600 units of fast-acting insulin<br><br>500 doses of metformin (sufficient for 166 patient days of treatment at a standard starting dose)<br><br>No sulphonylureas	Emergency medicine, surgical, or anesthetic background	Diabetes	Short acting insulin and oral hypoglycemics (if it could be demonstrated that patients had previously been prescribed pre-disaster)<br><br>Patients referred from the primary care/emergency section to the surgeons to determined the need for operation<br><br>Surgical management was by drainage and excisional debridement second daily<br><br>Wounds irrigated with potable water and dressed with simple gauze	Patients remained in hospital or were discharged home and returned for outpatient follow-up using the hospital's integrated patient transport<br><br>At the end of deployment, patients with ongoing wound or other medical issues were referred to a local regional medical center<br><br>Current inpatients in the field hospital at this time were directly transferred to other hospitals at the direction of the local Ministry of Health<br><br>Patients will be provided with up to 14-day	-222 operating theatre visits<br>-73 of the 222 (32.9%) OT visits and 30 of the 131 patients (22.9%) were diabetic<br>-The majority (26 of 30; 86.7%) of the diabetic cohort presented with a typhoon-related injury<br>-The proportion of surgical patients with diabetes was four times higher than the baseline prevalence of the disease<br>-Diabetic patients required more operations than their non-diabetic counterparts	Local medical supplies depleted quickly, and within days, people with diabetes found that they had no medication and limited means of getting renewed supply<br><br>The medication available was limited -> normal regimes were not available ->people had to change to unfamiliar drugs<br><br>Prescriptions were not necessarily available along with medical records -> so reliance was made on diabetic patients memorizing their regular medication regimes. Using interpreters to obtain medical history compounded this	Alignment with the national formulary of the disaster affected host nation is essential in pre departure preparation<br><br>Clear operational guidelines on field management and patient follow-up<br><br>Strong integration with Ministry of Health services in the relief and recovery phase<br><br>Improved medical record keeping and discharge summaries<br><br>Clinical staff must be familiar with the initiation and use of antidiabetic agents to which they may no longer be familiar<br><br>Development of a model for Acute	Focus on diabetes, no data are available on other NCDs<br><br>Methodology(Field report)-> Future strategies are needed to plan the validation of the model propose

MANUSCRIPT GENERAL INFORMATION				DISASTER		EMERGENCY MEDICAL TEAMS (EMTS)									NON COMMUNICABLE DISEASES (NCDs)			MAIN FINDINGS			
**1st Author/Year of publication**	**Title**	**Type of study**	**Aim**	**Disaster**	**Country/Year of occurrence**	**EMT type**	**EMT sending institution**	**EMT location**	**EMT staff**	**EMT capacity**	**Time of deployment/Operational time**	**Length of stay**	**Equipment (related to NCDs)**	**EMT training /background**	**NCDs of interest**	**Patients treatment**	**Other actions taken**	**Results**	**Challenges**	**Suggestions**	**Limitations of the study**
																Surgical management was by drainage and excisional debridement second daily until tissue appeared healthy and infection was controlled<br>Healing was obtained by secondary intention<br>Antimicrobial therapy: third-generation cephalosporin and metronidazole, with meropenem for refractory cases	supply of medications on discharge	(average 2.4 versus 1.5)<br>-All five major amputations (trans-tibial or trans-femoral) were performed in diabetics, as a consequence of advanced sepsis	uncertainty<br>Instituting new diabetic therapy with oral hypoglycemics was not possible from a perspective of medication supply and not considered safe given the difficulty with follow-up	Non-Communicable Disease Care Post Sudden Onset Disaster (Team composition and equipment, leadership and engagement, patient-centred care, data and medical records)	
van Berlaer G/2016	A refugee camp in the centre of Europe: clinical characteristics of asylum seekers arriving in Brussels	Retrospective descriptive cross-sectional study	To describe the demographic and clinical characteristics of asylum seekers who arrived in a huddled refugee camp (Syrian refugees), in the centre of a well developed country with all medical facilities (Brussels)	Refugees from 63 different countries, but most were from Iraq , Syria, Morocco, , Afghanistan and Palestine	Syria war/2015	Field Hospital (Médecins du monde)-> not WHO classifies	Médecins du monde	Refugees camp (Brussels)	400 certified physicians, nurses, pharmacists, logisticians and interpreters. MdM registered all volunteers and verified their diploma and licence to work in Belgium. An outpatient assistance team with a physician, a nurse and an interpreter provided on-the-spot healthcare for patients not able to leave their tents, or referred them to the field hospital for further care when necessary						Respiratory diseases,skin, digestive diseases,hypertension, diabetes, asthma, epilepsy, mental health issues	Registration on admission and basic triage in urgent and less urgent patients was performed by trained nurses-Each patient was subsequently interviewed and examined by physicians, with competent interpreters present during all clinical encounters.<br>Patients were treated on the spot if appropriate, vaccinated when	An outpatient assistance team with a physician, a nurse and an interpreter provided on-the-spot healthcare for patients not able to leave their tents, or referred them to the field hospital for further care when necessary Patients requiring emergency care, laboratory tests, medical imaging or hospitalisation were transferred to hospitals in the Brussels area. Patients				• The study included only patients self-presenting or referred by outpatient assistance teams to the field hospital in the autumn of 2015, preventing extrapolation to all asylum seekers, and other seasons.<br>• A number of diagnoses remained tentative, since laboratory and imaging tests were unavailable,<br>and patient anonymity prevented longitudina

MANUSCRIPT GENERAL INFORMATION				DISASTER		EMERGENCY MEDICAL TEAMS (EMTS)									NON COMMUNICABLE DISEASES (NCDs)			MAIN FINDINGS			
**1st Author/Year of publication**	**Title**	**Type of study**	**Aim**	**Disaster**	**Country/Year of occurrence**	**EMT type**	**EMT sending institution**	**EMT location**	**EMT staff**	**EMT capacity**	**Time of deployment/Operational time**	**Length of stay**	**Equipment (related to NCDs)**	**EMT training /background**	**NCDs of interest**	**Patients treatment**	**Other actions taken**	**Results**	**Challenges**	**Suggestions**	**Limitations of the study**
																needed and received explanations in their own language.	requiring follow-up were requested to re-present to the field hospital or referred to primary or dental care facilities, or newborn consultations, with referral letters				l follow-up. • The lack of uniform standards to collect symptoms and diagnoses makes comparison with other data sets challenging
Kevin KC Hung /2013	Disease pattern and chronic illness in rural China: The Hong Kong Red Cross basic health clinic after 2008 Sichuan earthquake	Cross-sectional records-based study	To identify the health needs and chronic disease prevalence of rural Chinese following a major earthquake	Earthquake	China, Sichuan earthquake, 2008	Hong Kong Red Cross (HKRC) basic healthcare clinic	Hong Kong Red Cross	Yanmen town, Jiangyou	The two HKRC medical teams composed of seven doctors, six nurses and one senior health coordinator, all providing basic healthcare to villagers		3 weeks after the earthquake				Musculoskeletal, respiratory ,gastrointestinal problems, high prevalence of hypertension				The management of chronic diseases was an important issue	The management of chronic diseases was an important issue, especially Medical responders need to be aware of the potential pre-existing disease burden in the community, with the possible exacerbation in post-disaster situations. Careful planning on the use of treatment guidelines with particular focus on the local health resources available and issues with continuation care will provide better care for the patients.	The most significant limitation is that there was no baseline data available to understand the effect of the earthquake on the health of villagers. The categorisation of disease was at the discretion of the doctor providing the consultation and might have suffered from reduced accuracy due to the limited diagnostic tests available and lack of standardised coding practice
Debarati Guha-Sapir/2007	Patterns of chronic and acute diseases after natural disasters – a study from the International Committee of	Cross-sectional, record-based study	To assess the pattern of diseases in the immediate aftermath after a major catastrophe	Indian Ocean tsunami	Aceh, Indonesia /2004	Red Cross field hospital	International Committee of Red Cross	Aceh				From January 15 to 31, 2004			Chronic diseases (43.5% of the the diagnosis): respiratory diseases, hypertension, diabetes, and acute manifestati					Chronic diseases underlie a substantial proportion of consultations of disaster-affected populations. Medical teams should be prepared for	

MANUSCRIPT GENERAL INFORMATION				DISASTER		EMERGENCY MEDICAL TEAMS (EMTS)									NON COMMUNICABLE DISEASES (NCDs)			MAIN FINDINGS			
**1st Author/Year of publication**	**Title**	**Type of study**	**Aim**	**Disaster**	**Country/Year of occurrence**	**EMT type**	**EMT sending institution**	**EMT location**	**EMT staff**	**EMT capacity**	**Time of deployment/Operational time**	**Length of stay**	**Equipment (related to NCDs)**	**EMT training /background**	**NCDs of interest**	**Patients treatment**	**Other actions taken**	**Results**	**Challenges**	**Suggestions**	**Limitations of the study**
	the Red Cross field hospital in Banda Aceh after the 2004 Indian Ocean tsunami														on of chronic diseases (e.g. asthma), Mental diseases were analysed separately					acute presentations of chronic illnesses. Children suffer mainly from infectious and other diseases in the same diagnostic groups as in an otherwise normal, poor population. Advantage should be taken of the presence of large numbers of medical and nursing volunteers and funds to not only rebuild but also improve the pre-disaster healthcare infrastructure in poor regions. Trauma and injury are not an important cause of morbidity among women or children, which most likely is attributed to survival bias Data from hospitals and public health agencies should be used to design future public health emergency interventions	
Fernald/2007	The Mobile Army Surgical Hospital Humanitarian Assistance Mission in Pakistan: The Primary Care Experience	Cross-sectional, record-based study	To describe the experiences during the deployment of a mobile army surgical hospital	Earthquake	Pakistan/ 8th October 2005	Mobile Army Surgical hospital (MASH)	American Government	Muzaffarabad, a city where 60% of the buildings had been destroyed in the earthquake	Surgery team implemented with two family medicine physicians, one paediatrician, and one internist	84-bed, surgically oriented unit, consisted of two intensive care units (ICUs), an intensive care ward, a minimal care ward, and two operating	21 October 2005/Primary care operated 7 days per week, 12 hours per day, for the entire deployment	4 months		Two medicine physicians, one paediatrician, and one internist augmented the assigned surgical	Chronic musculoskeletal disease, headache, gastroesophageal reflux, cerebrovascular accidents, renal failure, myocardial infarction,		Initially, all patients were seen in the emergency medicine section, and primary care providers were assigned to the emergency	Top five diagnoses were viral upper respiratory infections (40%), chronic musculoskeletal pain (28%), gastroes	After the first month of the mission, the surgical patient load decreased, whereas primary care increased to 90% of patient encounters. Before	- Military planners must recognize that primary care is essential<br>- Mission planning must include sufficient primary care personnel, medications, and	

MANUSCRIPT GENERAL INFORMATION				DISASTER		EMERGENCY MEDICAL TEAMS (EMTS)									NON COMMUNICABLE DISEASES (NCDs)			MAIN FINDINGS			
**1st Author/Year of publication**	**Title**	**Type of study**	**Aim**	**Disaster**	**Country/Year of occurrence**	**EMT type**	**EMT sending institution**	**EMT location**	**EMT staff**	**EMT capacity**	**Time of deployment/Operational time**	**Length of stay**	**Equipment (related to NCDs)**	**EMT training /background**	**NCDs of interest**	**Patients treatment**	**Other actions taken**	**Results**	**Challenges**	**Suggestions**	**Limitations of the study**
										rooms. As the mission progressed, a separate primary care section and outpatient clinic evolved to meet an overwhelming need for primary care.				personnel	respiratory failure		medicine team -> The volume of patients with chronic medical conditions and minor illnesses rapidly increased and, within 1 week, a triage tent was established to prioritise cases ->a separate section gradually developed through a series of innovative actions to constantly allow a limited number of primary care staff members to see a large number of outpatients in a surgically oriented hospital-> A fully functioning primary care clinic evolved eventually, with dedicated medics, nursing staff, immunisation section, phlebotomy services, and outpatient pharmacy	ophageal reflux (20%), scabies (13%), and headach (5%)	deployment -> There was anticipation of the need for primary care, and primary care providers were attached to the MASH. -> However, concern regarding digression from the surgical mission of the MASH prevented the establishment of a clear concise plan for primary care	supplies, with special emphasis on paediatric needs<br>Compassion fatigue and staff burnout must be anticipated. In prolonged missions, 1 day of rest per week for staff members are essential Translators are especially vulnerable to mental fatigue<br>- Working closely with local health care providers and NGOs is essential for maintaining a uniform standard of health care<br>- Military units should work through the local system and advises against inventing a new system with Western standards that the population cannot sustain<br>- The Pakistani physicians proved invaluable resources regarding local medical customs, local languages, local treatments, referral locations, available resources, and patient expectations of medical care	
